# Tumor Infiltrating Neutrophils Are Enriched in Basal-Type Urothelial Bladder Cancer

**DOI:** 10.3390/cells9020291

**Published:** 2020-01-25

**Authors:** Giulio Eugenio Mandelli, Francesco Missale, Debora Bresciani, Luisa Benerini Gatta, Patrizia Scapini, Elena Caveggion, Elisa Roca, Mattia Bugatti, Matilde Monti, Luca Cristinelli, Sandra Belotti, Claudio Simeone, Stefano Calza, Laura Melocchi, William Vermi

**Affiliations:** 1 Department of Molecular and Translational Medicine, School of Medicine, University of Brescia, 25125 Brescia, Italy; g.mandelli@studenti.unibs.it (G.E.M.); f.missale@studenti.unibs.it (F.M.); deborabresciani96@gmail.com (D.B.); luisa.benerinigatta@unibs.it (L.B.G.); bgtmtt@hotmail.it (M.B.); m.monti002@unibs.it (M.M.); 2 IRCCS Ospedale Policlinico San Martino, 16121 Genova, Italy; 3 Department of Otorhinolaryngology, Head and Neck Surgery—University of Genoa, 16121 Genova, Italy; 4 Department of Medical and Surgical Specialties, Radiological Sciences, and Public Health, University of Brescia, 25100 Brescia, Italy; elisaroca@gmail.com (E.R.); lcristinelli@gmail.com (L.C.); belot.sandra@gmail.com (S.B.); claudio.simeone@unibs.it (C.S.); 5 Section of General Pathology, Department of Medicine, University of Verona, 37134 Verona, Italy; patrizia.scapini@univr.it (P.S.); elena.caveggion@univr.it (E.C.); 6 ASST Spedali Civili di Brescia, 25100 Brescia, Italy; 7 Unit of Biostatistics, Department of Molecular and Translational Medicine, University of Brescia, 25125 Brescia, Italy; stefano.calza@unibs.it; 8 Department of Medical Epidemiology and Biostatistics, Karolinska Institutet, 17177 Stockholm, Sweden; 9 Department of Pathology, Fondazione Poliambulanza, 25100 Brescia, Italy; laura.melocchi@gmail.com; 10 Department of Pathology and Immunology, Washington University School of Medicine, St. Louis, MO 63130, USA

**Keywords:** tumor-associated neutrophils, bladder cancer, basal, CD66b, CD3

## Abstract

Background: Urothelial bladder cancers (UBCs) are distinct in two main molecular subtypes, namely basal and luminal type. Subtypes are also diverse in term of immune contexture, providing a rationale for patient selection to immunotherapy. Methods: By digital microscopy analysis of a muscle-invasive BC (MIBC) cohort, we explored the density and clinical significance of CD66b^+^ tumor-associated-neutrophils (TAN) and CD3^+^ T cells. Bioinformatics analysis of UBC datasets and gene expression analysis of UBC cell lines were additionally performed. Results: Basal type BC contained a significantly higher density of CD66b^+^ TAN compared to the luminal type. This finding was validated on TCGA, GSE32894 and GSE124305 datasets by computing a neutrophil signature. Of note, basal-type MIBC display a significantly higher level of chemokines (CKs) attracting neutrophils. Moreover, pro-inflammatory stimuli significantly up-regulate CXCL1, CXCL2 and CXCL8 in 5637 and RT4 UBC cell lines and induce neutrophil chemotaxis. In term of survival, a high density of T cells and TAN was significantly associated to a better outcome, with TAN density showing a more limited statistical power and following a non-linear predicting model. Conclusions: TAN are recruited in basal type MIBC by pro-inflammatory CKs. This finding establishes a groundwork for a better understanding of the UBC immunity and its relevance.

## 1. Introduction

Urothelial bladder cancer (UBC) is the ninth most common cancer worldwide [1]. Despite that 80% of cases are limited to the superficial layers of the urothelium (non-muscle invasive bladder cancer, NMIBC) [2], about 20% display a muscle-invasive aggressive disease (MIBC) at the onset and a third of them will eventually develop metastasis over time [3]. Radical cystectomy with lymph-node dissection represents the standard of treatment for operable MIBC; however, 5-year overall survival (OS) is 50% or even lower in locally advanced disease [4]. As for unresectable and metastatic MIBC, the treatment algorithm is rapidly evolving with the recent approval of immunotherapy beyond the first line Platinum-based chemotherapy [5], the latter ensuring a five-year OS of only 13–22% [6]. Nevertheless, only about 23–26% of patients show objective response with second line immunomodulatory agents [7], indicating that a better patient selection is mandatory.

Recent advances in the understanding of the genomic and transcriptomic landscape of UBC have proposed additional windows of intervention based on tumor-cell intrinsic molecular features. Specifically, gene expression profile analysis revealed that UBC is an extremely heterogeneous disease that can be grouped into two main subtypes, namely basal and luminal type [8]. Molecular pathways controlling tumor growth are divergent in basal vs. luminal UBC, thus providing the rationale for their different biological behavior [9]. Luminal type UBCs display a hyperplastic well differentiated papillary histology at the onset and are frequently enriched with activating mutations in HRAS and fibroblast growth factor receptor 3 (FGFR3) [10]. This subgroup could benefit by FGFR3 targeting, recently approved also in the neoadjuvant setting [11]. Basal type UBCs originate instead from flat high-grade precursors including carcinoma in situ (CIS) via inactivation of TP53, RB1 and hyper-activation of the transcription factors STAT3, NFKB1 and TP63 in the stem cells of the basal layer. These features might account for the more aggressive behavior of basal type UBCs [12].

Beyond tumor-cell intrinsic molecular features, the UBC immune contexture can also yield information relevant to prognosis and to treatment response, particularly to immunotherapy [13]. Of note, luminal and basal subtypes display a different microenvironment in term of immune and stromal cells [14,15,16]; moreover, the recently identified “infiltrated” subtypes display a specific immunological signatures [17,18]. Basal type UBCs show a higher level of immunogenicity based on a set of observations. Firstly, high grade CIS response to intravesical instillation of Bacillus Calmette-Guérin (BCG) is highly dependent on immune contexture [19,20]. Secondly, despite some controversial data, a high density of tumor infiltrating lymphocytes (TILs) in basal type MIBCs is correlated to a better outcome [15,21]. Thirdly, basal-type MIBCs displaying a high density of immune infiltration respond better to adjuvant chemotherapy [15], in contrast to luminal-type MIBC that do not derive much benefit [22]. However, it should be reminded that a recently identified subset of ‘luminal-infiltrated’ may respond to immunotherapy [23].

Various immunosuppressive mechanisms affect adaptive T-cell response to cancer, such as the well-known PD-1/PD-L1 axis, thus enhancing tumor growth and progression [24]. This has set the rationale for PD-1/PD-L1 blockade, with promising results also in patients with advanced UBC progressing after platinum-based chemotherapy [25]. At present, PD-L1 expression both on tumor cells and infiltrating immune cells is the only accepted predictor of response in UBC [6,25], although with a still limited performance in the selection of responders [7,26]. Some evidence shows that the tumor mutational burden (TMB), a CD8^+^ T cell signature, a TGF-β immune suppressive milieu and the UBC molecular subtype can predict response to immune check-point inhibitors (ICI) as well, but a reliable predictive nomogram is still lacking. Notably, the basal and the luminal-infiltrated subtypes have shown the best benefit from ICI compared with other subtypes [23,26], but only 20% of patients reached a complete response [5].

All these findings suggest that other immune escape mechanisms might be in place in MIBC. Among the latter, infiltration by myeloid cells might account for a reduced specific T-cell response. Granulocyte population found within the tumor tissues are referred as tumor-associated neutrophils (TANs) [27]. In mouse, TANs can be polarized in vitro [28,29]. N1-type TANs exert anti-tumor functions, whereas N2-type TANs are TGF-β-driven and sustain cancer progression. It is still unknown whether human TANs display similar polarization. The most recent literature considers neutrophils as a population with potentially pro-inflammatory or anti-inflammatory functions showing either a pro-tumoral or an anti-tumoral program, thus partially overcoming the dichotomous definition of N1 and N2 cells [30]. Data from large retrospective cancer cohorts [31] have supported a pro-tumor function of TANs, with a high TAN tumor density significantly associated to a worse outcome [32]. However, emerging data have challenged this view showing TANs with anti-tumor activities [33]. Numerous works have proposed a clinical utility of the peripheral neutrophil to lymphocytes ratio (NLR) in bladder cancer in term of prognosis [34] and recurrence [35] for MIBCs who underwent cystectomy. On the contrary, works on TAN and UBCs are very limited. Elevated TANs have been correlated with poor prognosis [36], whereas a lower TANs density predict a better response to adjuvant chemotherapy [37]. However, studies adherent to analytical standards of immunoscore evaluation of TAN density, are still lacking [38].

In this study, we have measured the TAN and TIL density in a retrospective cohort of MIBC by applying digital microscopy to CD66b and CD3 stained whole sections. Our findings suggest that high density of CD66b^+^ TAN represent a distinct feature of basal type MIBC. Moreover, based on in silico analysis of UBC datasets and in vitro studies on UBC cell lines, we found that TAN-attracting chemokines are enriched in basal type UBC. These findings might be of help in patient selection to established and novel immunotherapy approaches.

## 2. Materials and Methods

### 2.1. Patient’s Cohort

This retrospective study was conducted in compliance with the Helsinki Declaration and with policies approved by the Ethics Board of ASST Spedali Civili di Brescia (IRB code: NP 2483/2016 to WV). The cohort included previously untreated patients affected by muscle invasive bladder cancer (MIBC), treated by cystectomy from 2006 to 2016 in the Department of Urology, ASST Spedali Civili di Brescia (Brescia, Italy). Surgical specimens were reviewed and staged according to the TNM staging system [39]. Exclusion criteria included any treatment before diagnostic biopsy and occurrence of other malignancies. Formalin fixed paraffin-embedded (FFPE) UBC were retrieved from the archive of the Department of Pathology, ASST Spedali Civili di Brescia (Brescia, Italy). Clinical follow-up data were retrieved from the Department of Urology, ASST Spedali Civili di Brescia (Brescia, Italy).

The analyzed cohort consisted of 84 MIBCs (T category ≥ pT2); classification of MIBCs into luminal and basal subtypes is described later. Demographic, histological and clinical findings are summarized in Supplementary Table S1.

### 2.2. Immunohistochemistry

Four-micron thick FFPE sections were used for immunohistochemical staining. Heat mediated antigen retrieval was performed in a microwave oven and endogenous peroxidase activity was quenched using 0.3% hydrogen peroxide (Sigma-Aldrich, Saint-Louis, MO, USA) diluted with methanol (Sigma-Aldrich, Saint-Louis, MO, USA). After washing with Tris-Buffered Saline (TBS, Sigma-Aldrich, Saint-Louis, MO, USA) solution, slides were incubated with the primary antibody for 1 h at room temperature and revealed by a 30 min incubation with a horseradish-peroxidase polymer (Novolink Polymer Detection System - Leica Biosystems, Wetzlar, Germany), followed by 3,3′-diaminobenzidine (Leica Biosystems, Wetzlar, Germany) as chromogen. Sections were counterstained with Mayer’s haematoxylin (Bioptica). For double immunohistochemical staining, after completing the first immune reaction, the second was visualized using Mach 4 MR-AP (Biocare Medical, Pacheco, CA, USA), followed by Ferangie Blue (Biocare Medical, Pacheco, CA, USA) as chromogen.

For immunohistochemistry (IHC), primary antibody include: anti-CD66b (clone G10F5, Mouse IgM, dilution 1:120, BioLegend, San Diego, CA, USA), anti-CD3 (clone SP7, Rabbit IgG, dilution 1:100, Thermo Fisher Scientific, Waltham, MA, USA), anti-CD11b (polyclonal, Rabbit, dilution 1:300, Sigma Aldrich, Saint-Louis, MO, USA), anti-CD15 (clone MMA, Mouse IgM, dilution 1:400, BD Biosciences, San Jose, CA, USA) and anti-Arginase-1 (clone SP156, Rabbit IgG, dilution 1:100, Cell Marque, Rocklin, CA, USA).

The classification of MIBCs into subtypes was performed on TURB using a four marker panel composed of CK5/6 (basal marker), CK14 (basal marker), CK20 (luminal marker) and UPK2 (luminal marker), as previously described [40]; similarly, detailed for pSTAT3 and FOSL1 stain are reported in our previous study [40].

### 2.3. Digital Microscopy

Stained slides were acquired using the Aperio CS2 digital scanner and ScanScope software (Leica Biosystems, Wetzlar, Germany). Images were viewed and organized using ImageScope software (Leica biosystems, Wetzlar, Germany). Each scanned image was annotated manually and IHC Nuclear Image Analysis algorithm was chosen for the analysis. The whole tumor area has been considered for the analysis, with the exclusion of necrotic areas. Data are expressed as absolute number of CD66b^+^ or CD3^+^ cells *per* mm^2^.

### 2.4. Statistical Analysis

For histological, clinical and pathological analysis, the qualitative variables were described as absolute and relative frequencies; standard descriptive statistics were used for continuous variables, expressing means, medians, interquartile ranges (IQR) and standard deviations. Correlation between cell population densities were computed using Spearman correlation coefficient. Shapiro–Wilk test was applied, normality distribution of continuous variables (immune cells densities) was not confirmed and non-parametric test were subsequently used.

The main survival endpoint was the overall survival (OS), defined as the time between the date of the cystectomy and the date of death; progression free survival (PFS), as secondary endpoint, was defined as the time between the date of the surgery and the date of recurrence. In the absence of an event, survivals were censored at last follow-up visit. Qualitative variables were compared between groups using Fisher exact test and quantitative variables by Mann-Whitney or Kruskal-Wallis test (followed by p-values adjustment by Dunn’s test for multiple comparisons). Cut-offs in continuous predictors for the definition of groups rich (Hi) or poor (Lo) of immune cells were set at the median value for each distribution. A tissue neutrophil to lymphocyte ratio (tNLR; CD66b/CD3 counts) obtained for each sample was also computed as a variable. Univariate and multivariate survival analyses were performed using Cox proportional hazard models; multivariable models were built by a backward selection applying Likelihood-ratio test and Akaike information criterion; estimates were reported as hazard ratio (HR) with 95% Confidence Intervals (CI95%). Univariate survival curves were plotted by the Kaplan-Meier method with covariates adjustment and compared by the Log-rank test.

Survival multivariable models were subsequently built including immune cells densities as continuous variables, after log transformation; to account for potential non-linear effects on HR penalized splines with degrees of freedom selected via Corrected Akaike’s Information Criterion (AICc, [41]) were used.

For in vitro experiments, UBC cell lines were analyzed using the One-way ANOVA with Bonferroni’s correction.

In all analysis a two-tailed *p* value < 0.05 was considered significant. GraphPad Prism (San Diego, CA, USA), Stata (version 13.0, College Station, TX, USA) and R (version 3.5.1) were used for statistical analysis.

### 2.5. Cell Cultures

RT4 (ATCC^®^ HTB2TM) and 5637 (ATCC^®^ HTB9TM) cell lines were obtained from ATCC-LGC Standards Repository (Rockville, USA). 5637 cells were maintained in ATCC-formulated RPMI1640 Medium (cat. No. A10491-01, GibcoTM for Life Technologies - Thermo Fisher Scientific, Waltham, MA, USA). RT4 cells were maintained in ATCC-formulated McCoy’s 5a Medium Modified (cat. no. 26600-023, GibcoTM for Life Technologies - Thermo Fisher Scientific, Waltham, MA, USA). All media were supplemented with 10% fetal bovine serum (FBS) (cat. no. S0115, Biochrom, Berlin, Germany), 1% Penicillin/Streptomycin (cat. No. 15070-063, GibcoTM), and the cells were cultured at 37 °C and 5% CO_2_.

One hundred and forty-four-hour STAT3-silenced 5637 cells, as well as untreated 5637 cells and RT4 cells, were stimulated with a cocktail of recombinant human TNF-a (20 ng/mL; cat. no. 300-01A), recombinant human IL6 (20 ng/mL; cat. no. 200-06), and recombinant human IL1b (20 ng/mL; cat. no. 200-01B; all from PeproTech, EC, Ltd., London, UK) for 4 h or 24 h.

### 2.6. Neutrophil Isolation and Transwell Migration Assay

Circulating neutrophils were isolated from healthy donors by density gradient centrifugation (Ficoll-Paque; GE Healthcare Life Sciences) of whole blood and further purified by negative selection using the EasySep neutrophil enrichment kit (StemCell Technologies, Vancouver, BC, Canada) as previously described [42]. The purity of isolated neutrophils was > 99.8%, as determined by flow cytometry. Neutrophil direct migration (chemotaxis) was measured in Transwell chamber (3 μm; Corning Costar), as previously described [43]. Briefly, 100 μL of neutrophil suspensions (2 × 10^6^/mL) were added to the top chambers, whereas 600 μL of control medium or tumor-conditioned supernatants from luminal-type RT4 or basal-type 5637 UBC cell lines, either unstimulated or treated with the pro-inflammatory cytokine cocktail (TNF-a, IL6 and IL1b) for 4 or 24 h (as described above) were added to the bottom wells. After 45 min, the plates were spun, the inserts were removed, and the number of migrated cells were counted with CyQuant cell proliferation assay kit (Invitrogen SRL). Parallel samples were included to determine the signal intensity from the total cell number loaded into the Transwell inserts.

### 2.7. Quantitative RT-PCR (qRT-PCR)

CXCL1, CXCL2, CX3CL1 and CXCL8 mRNA targets were quantified by reverse transcription-polymerase chain reaction (qRT-PCR) assay using the Vii-A 7 Real-Time PCR System (Applied Biosystems, Thermo Fisher Scientific, Waltham, MA, USA). Total RNA was extracted from UBC cells by TRIzol™ (cat. no. 15596026, Invitrogen™, Thermo Fisher Scientific). The cDNA was synthesized by iScript gDNA cDNA Synthesis kit (cat. no. 1725035, Bio-Rad Laboratories Inc., Hercules, CA, USA) from 1 µg of total RNA, in a total volume of 20 µL. One µL of the cDNA synthesis reaction was used for the specific amplification of the target transcripts. The Hypoxanthine-guanine phosphoribosyltransferase 1 (HPRT1) transcript was used as normalization control. The PCR was performed in a total volume of 20 µL with TaqMan^®^ Universal Master Mix II (cat. no. 4369016, Applied Biosystems, Thermo Fisher Scientific) and the Gene Expression Assay (Supplementary Table S2). The threshold cycle (Ct) was determined for each sample and quantification was performed using the comparative Ct method. ΔCt was derived as Ct_Target_ – Ct_Housekeeping_ and considered for statistical analysis.

### 2.8. STAT3 Silencing

STAT3 (Genbank accession n° NM_139276.2, NM_213662.1, NM_00315.3) and control knockdown was obtained using specific-STAT3 siRNA (assay ID S744) and control scrambled-siRNA (ca. no. 4390846), obtained from Ambion (Thermo Fischer Scientific, Waltham, MA, USA). Validation of si-STAT3 silencing was obtained as previously described. For more details see Benerini Gatta L. et al. 2019 [40].

### 2.9. Data Preprocessing and Statistical Analysis of the TCGA, GSE32894 and GSE124305 Datasets

Raw counts for primary solid tumor samples were downloaded from GDC portal harmonized repository using TCGAbiolinks R/Bioconductor package (*n* = 408 cases). The FFPE samples were removed. The duplicated samples counts were averaged (*n* = 388). The library size normalization factors were obtained with the trimmed mean of M-values (TMM) [44] and gene expression was computed as log2-RPKM. GSE32894 and GSE124305 expression values were preprocessed applying a quantile normalization and transformed on log scale.

TCGA and GSE32894 include chemotherapy-naive MIBC [17,18], whereas GSE124305 include MIBC cystectomy samples obtained after Neoadjuvant Chemotherapy treatment and containing residual tumor. As reported [45], basal and luminal subtypes from this post-treatment cohort express a gene signatures consistent with a basal and luminal phenotype comparable to their matched pre-treatment tumor samples [22]. In all datasets, we have considered only MIBC cases. Moreover, in addition to basal and luminal subtypes, each of three datasets includes other molecular subtypes based on their immune signature, defined by consensus clustering in each of the three independent cohorts.

Global gene signature (Chemokine or PMN) were computed using single sample gene enrichment based on Gene Set Variation Analysis (GSVA) [46]. The neutrophil signature was obtained from the study by Newman et al. [47] (Supplementary Table S3). Chemokines with pro-inflammatory functions have been defined based un published classifications [48] (Supplementary Table S3).

Differential expression among subtypes at gene level for both signatures was evaluated using generalized linear modelling [49]. Specifically for TCGA data, negative binomial generalized log-linear models were applied to read counts while for GSE datasets, ordinary least square models, with moderated t-statistics computation [50].

Unsupervised hierarchical clustering was performed on expression values using Euclidean distance and Ward method [51].

A supervised classification algorithm was applied on the three datasets independently to ascertain the potential predictive performance of selected signatures on subtypes prediction. We adopted a Random Forest (RF) algorithm and estimated its performance based on Out-Of-Bag (OOB) error rate. In order to visualize the estimated relationship between samples, we used the proximity matrix derived from RF in a hierarchical clustering.

## 3. Results

### 3.1. Patient Characteristics

The clinical and pathological findings of the MIBC patient cohort are summarized in Table 1 and detailed in Supplementary Table S1. The median age was 72 at surgery. Median follow-up time was 60 months (CI95% 36; 84). Median overall survival was 65 months (CI95% 25; 92), whereas median progression free survival was 27 months (CI95% 10; ∞). During follow-up, 38 patients had recurrence and 38 patients died. None of the patients received neo-adjuvant chemo- or immunotherapy. After cancer recurrence, none of the patients received additional treatment. The univariate analysis confirmed the prognostic significance of the well-known clinical variables as higher age (*p* = 0.024), female gender (*p* = 0.003), higher pT category (*p* = 0.003), presence or nodal disease (N+) (*p* = 0.016), and higher Overall Stage (*p* = 0.002) associated with a worse OS; moreover, a higher pT category (*p* = 0.005), presence or nodal disease (N+) (*p* = 0.015) and higher Overall Stage (*p* = 0.005) were associated with a worse PFS (Table 2). In the cohort, despite the TNM staging system was confirmed to stratify prognosis, 23% of patients in stage II experienced recurrence.

### 3.2. CD66b^+^ TAN and CD3^+^ T Cell Immune-Contexture in Muscle Invasive UBC

**Figure 1:**
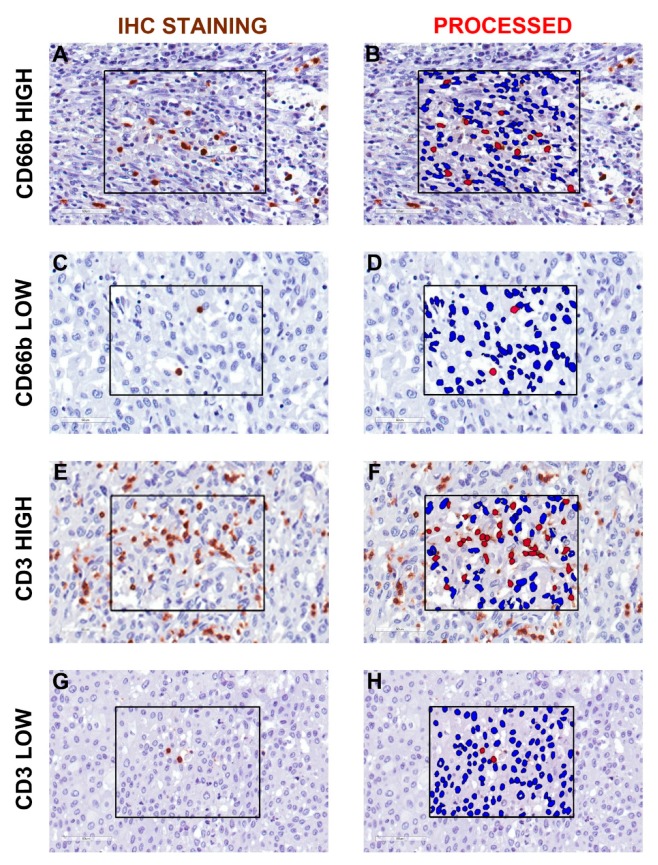
Representative image analysis for CD66b^+^ TAN and CD3^+^ T cells in human MIBC. Sections are from four representative human MIBC (case #66, #31, #7 and #46) and stained as labeled and counterstained with hematoxylin (**A**,**C**,**E**,**G**). On the right column, the corresponding processed area is shown (**B**,**D**,**F**,**H**). Case with high and low density of CD66b^+^ TAN (**A** and **C,** respectively) and CD3^+^ T cells (**E** and **G,** respectively) are displayed. Images acquired from digital slides using 400× magnification have been digitally resized using Adobe Photoshop.

**Figure 2:**
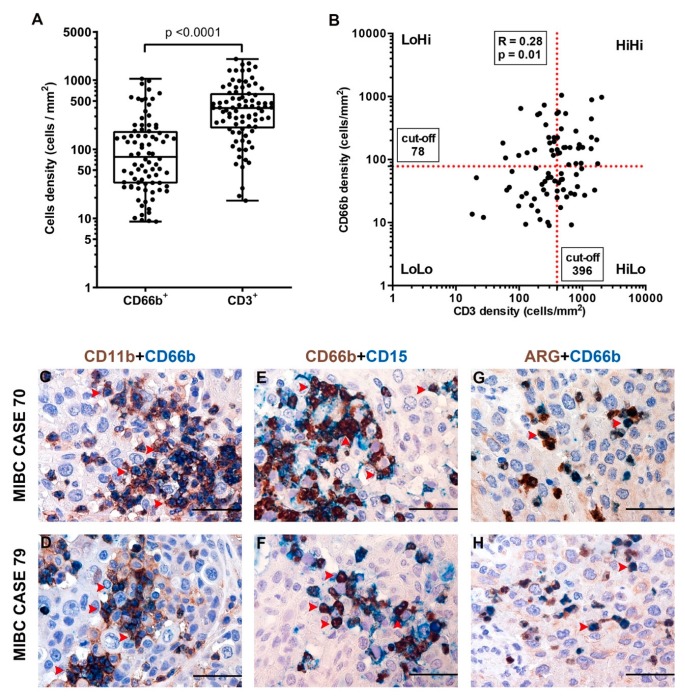
Density of CD66b^+^ TAN and CD3^+^ T cells and phenotype of TAN in human MIBC. Dot plots indicating the density of CD66b^+^ TAN and CD3^+^ T cells in the MIBC cohort is reported in (**A**); the correlation between the two variables is illustrated in (**B**). Sections are from two representative MIBC cases (case #70 in **C**, **E** and **G**; case #79 in **D**, **F** and **H**) and stained as labeled, illustrating CD66b^+^ TAN co-expressing neutrophil markers: CD11b, CD15 and ARG. Sections are counterstained with hematoxylin. Magnification 400× (scale bar 50 micron).

**Figure 3:**
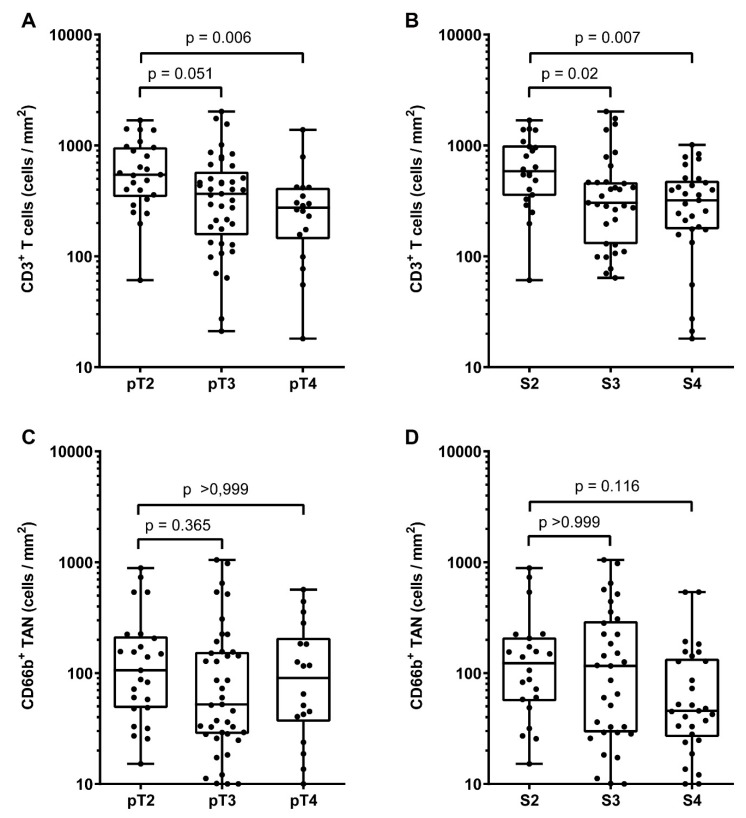
Clinical correlation of CD66b^+^ TAN and CD3^+^ T cells densities in human MIBC. Dot plots showing CD3^+^ T cells (**A**,**B**) and CD66b^+^ TAN (**C**,**D**) density distributions among different pT categories and Overall Stages in the MIBC cohort. *p* values were estimated by Kruskal-Wallis test and adjusted for multiple pairwise comparisons by Dunn’s method.

Data on the immune contexture of UBC are scant and limited to the lymphocytic infiltrate, while myeloid cells infiltrate has been less characterized. To expand this field, we quantified the density of CD3^+^ T cells and CD66^+^ TANs in the same retrospective cohort of muscle-invasive UBC (MIBC, *n* = 84) (Table 1, Table 2). Complete clinical, pathological and image analysis details of the cohort are reported in Supplementary Tables S1 and S4. A representative tissue block of the tumor was used for the analysis and representative images are shown in Figure 1A–H. A mean tumor area of 153.7 mm^2^ (range from 7.4 mm^2^ to 420.7 mm^2^) was obtained. The median density of CD66b^+^ TANs was 78 cells/mm^2^, whereas for CD3^+^ T cells the median density was 396 cells/mm^2^ (Figure 2A). The density of the two populations showed a significant correlation (R_S_ = 0.28, *p* = 0.011) (Figure 2B). Based on double stain performed on a set (*n* = 5) of MIBC, CD66b^+^ co-expressed neutrophils markers including CD11b, CD15 and arginase (Figure 2C–H). When compared with pathological and clinical features, we found that the density of the CD3^+^ T cells significantly decreased over UBC progression in term of pT category and Overall Stage (Figure 3, Table 3), whereas no correlations where observed for CD66^+^ TAN density. No significant associations were found between tissue NLR (tNLR) and clinical variables (Table 3).

### 3.3. Clinical Significance of CD66b^+^ TAN and CD3^+^ T Cell Immune-Contexture in MIBC

The clinical significance of the MIBC immune contexture was tested in term of overall survival (OS) and progression-free survival (PFS). The cut-offs points of low and high densities of CD3^+^ T cells and CD66b^+^ TAN were set at the median value for each distribution, being 396 cells/mm^2^ for CD3^+^ T cells and 78 cells/mm^2^ for CD66b^+^ TAN.

**Figure 4:**
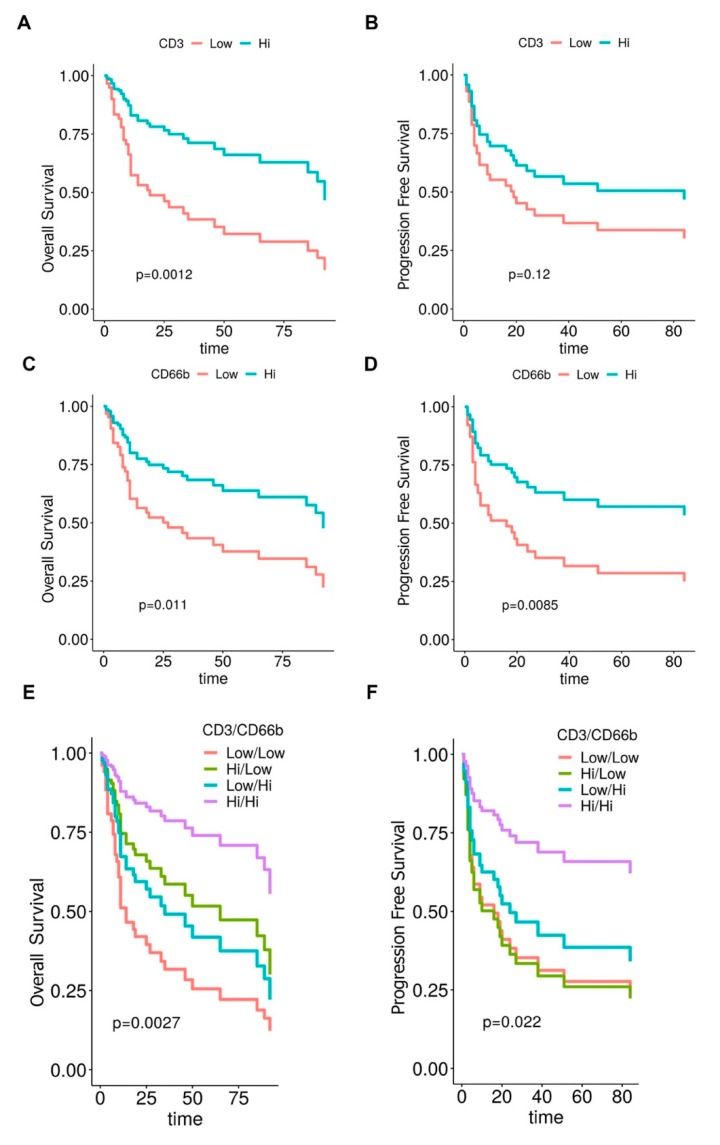
Clinical significance of CD66b^+^ TAN and CD3^+^ T cells in human MIBC. Kaplan-Meier curves illustrate the overall (**A**, **C** and **E**) and progression-free (**B**, **D** and **F**) survival in the MIBC cohort. *p* values are estimated by log-rank test and weighted for pT category (pT2/pT3-4).

Of note, a high density of both populations was associated to a better outcome at univariate analysis (Table 2), as also illustrated by Kaplan Meyer curves, weighted for pT category (Figure 4A–D). Based on these findings we further extended our analysis by defining a combined immunoscore. Notably, the subgroup of CD3^HIGH^CD66b^HIGH^ resulted in the best OS and PFS, whereas the subgroup of CD3^LOW^CD66b^LOW^ showed the worst outcome, as indicated by Kaplan Meyer curves analysis weighted for pT category (Table 2 and Figure 4E–F). By a backward selection of covariates, we subsequently devised the optimal Cox multivariable models for OS and PFS. Remarkably, the immunoscore resulted significant as prognostic factor for both OS and PFS (Table 4) and its removal resulted in a significant worsening of the performance of each model both for OS (*p* < 0.001) and PFS (*p* = 0.02) applying the Likelihood-ratio test.

We also investigated the effect of immune cells density variations on the HR along the whole range of CD3^+^ T cells and CD66b^+^ TAN values. Accordingly, Cox models for OS and PFS included immune cells densities as continuous log-variables modelled by penalized splines (Supplementary Figure S1). Notably, the effect of CD3^+^ T cells as favorable biomarker was confirmed, following a linear model for both OS and PFS prediction; on the contrary, the effect of CD66b^+^ TAN population for OS display a non-linear shape. Specifically, for CD66b, the highest risk is found around the second quartile, whereas the lowest one is identified at both tails. This finding weakens the inference of the results obtained by categorizing the CD66b* TAN density as variable for OS prediction.

### 3.4. A Higher Density of CD66b^+^ TAN Is Restricted to Basal Type MIBC Expressing the Transcription Factor STAT3

**Figure 5:**
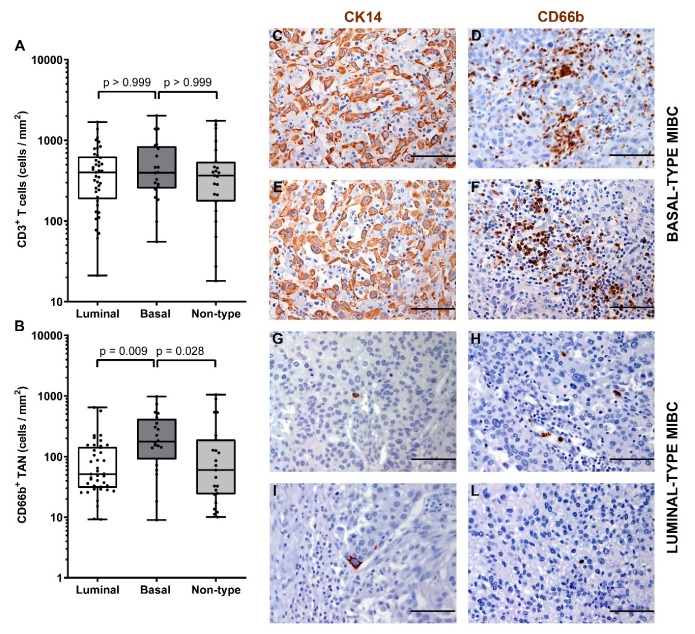
Density of CD66b^+^ TAN and CD3^+^ T cells in basal and luminal MIBC. Dot plots indicating the density of CD3^+^ T cells (**A**) and CD66b^+^ TAN (**B**) in the MIBC cohort subdivided by molecular subtypes. Sections from two basal-type MIBC cases (case 70 and 49, in **C**–**F**) and two luminal-type MIBC cases (case 25 and 34, in **G**–**L**) are stained as labeled and counterstained with hematoxylin; magnification 200× (scale bar 100 micron). *p* values were estimated by Kruskal-Wallis test and adjusted for multiple pairwise comparisons.

The immune contexture of the molecular subtype of MIBC has been poorly investigated. Data from previous studies indicate that luminal subtype is more likely correlated to a poor immune infiltrate, while basal tumors have high amount of infiltrated immune cells [14,15,16]. We sub-grouped our MIBC cohort in luminal-type, basal-type and non-type and analyzed the density of CD66b^+^ TAN and CD3^+^ T cells. We first classified the UBC cases on TURB (also validated on cystectomy tissue blocks) using a set of validated IHC markers including CK5/6, CK14, CK20 and UPK2 [52]. Based on this approach, the study cohort was composed of forty-two (47%) luminal-type UBC, 21 (25%) basal-type UBC and 26 (28%) “non-type” UBC. Notably, basal type MIBCs showed a significantly (*p* = 0.013) higher density of CD66b^+^ TAN compared to luminal and non-type MIBCs (Figure 5 and Table 3). On the contrary, no differences were observed in term of CD3^+^ T cells density (Figure 5 and Table 3).

**Figure 6:**
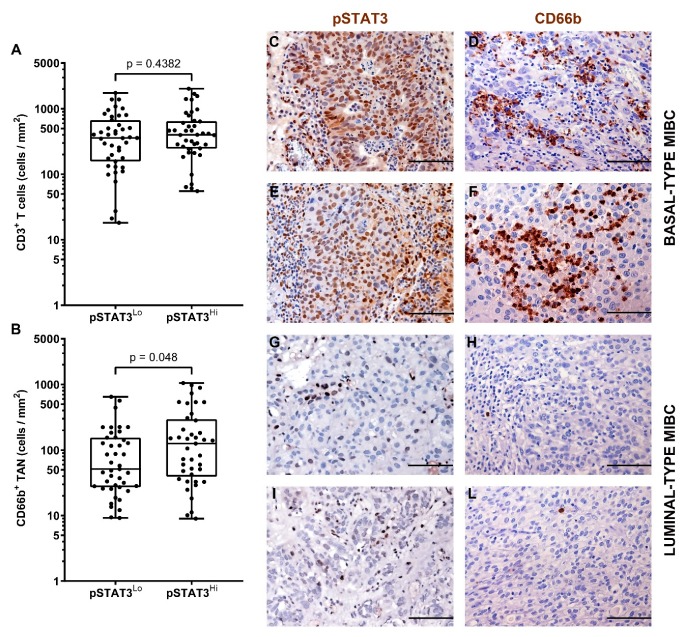
Density of CD66b^+^ TAN and CD3^+^ T cells in human MIBC with hyper-activation of STAT3. Dot plots indicating the density of CD3^+^ T cells (**A**) and CD66b^+^ TAN(**B**) in the MIBC cohort based on pSTAT3 expression. Sections are from two pSTAT3 HIGH (case 49 and 2, in **C**–**F**) and two pSTAT3 LOW (case 68 and 57, in **G**–**L**) MIBC cases and stained as labeled and counterstained with hematoxylin. Magnification: 200× (**C**–**L**), scale bar 100 micron. *p* values were estimated by Kruskal-Wallis test.

**Figure 7:**
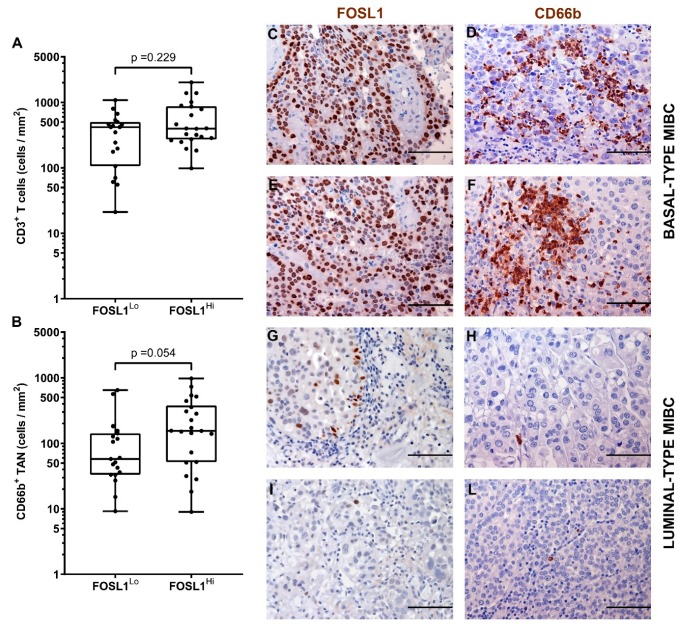
Density of CD66b^+^ TAN and CD3^+^ T cells in MIBC in relation to FOSL1 expression. Dot plots indicating the density of CD3^+^ T cells (**A**) and CD66b^+^ TAN (**B**) in the MIBC cohort based on FOSL1 expression. Sections are from two FOSL1 HIGH (case 49 and 14, in **C**–**F**) and two FOSL1 LOW (case 68 and 23, in **G**–**L**) MIBC cases and stained as labeled and counterstained with hematoxylin. Magnification: 200× (**C**–**L**), scale bar 100 micron. *p* values were estimated by Kruskal-Wallis test.

It has been recently reported that the hyper-activation of the transcription factor STAT3 is central in the transformation and progression of basal type UBC [53]. We also contributed to this field by showing that the expression of pSTAT3 and downstream targets is enriched in basal type MIBC [40]. Of note, we found that pSTAT3^+^ MIBC (score 2 and 3) were significantly more infiltrated by CD66b^+^ TAN, whereas no differences were observed in term of CD3^+^ T cells infiltration (Figure 6 and Table 3). We have recently shown that FOSL1 expression is strongly induced in basal-type UBC showing hyper-activation of STAT3 [40]. Moreover, among FOSL1-regulated genes, CXCL8 is also included [40]. It has been recently proposed that a FOSL1 signature including TAN-attracting CKs (CXCL8, CXCL6 and CXCL5) is activated in Basal B type breast cancer cell lines [54]. We found that the density of CD66b^+^ TAN slightly correlates with FOSL1 expression (Figure 7 and Table 3).

**Figure 8:**
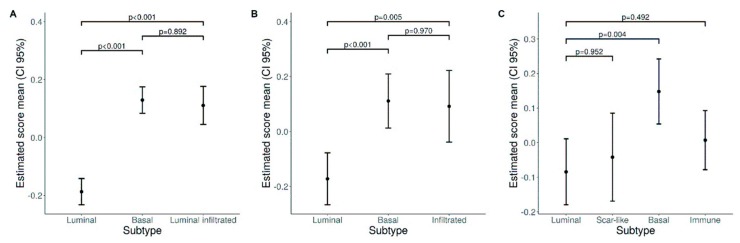
Neutrophil gene signature in UBC datasets. Plots show estimated mean score values and corresponding 95% confidence intervals for neutrophil GSE scores in bladder subtypes (**A** TCGA, **B** GSE32894, **C** GSE124305). Pairwise comparisons p-values were computed from linear models and adjusted for multiple comparisons.

We expanded our analysis in silico and tested the expression of a neutrophil gene signature (Figure 8 and Supplementary Figure S2–S4) in public available datasets on the transcriptome analysis of large UBC cohorts. To this end we started with the TCGA RNA-seq dataset [17]. Clustering analysis showed that genes belonging to a neutrophil signature (obtained from [47]) were significantly over-expressed in basal type and luminal-infiltrated MIBC, compared to luminal type (Figure 8A and Supplementary Figure S2). Applying the same procedure to additional independent datasets GSE32894 [18] and GSE124305 [45], we confirmed the enrichment of the neutrophil signature in basal type and infiltrated type MIBC (Figure 8B–C and Supplementary Figures S3 and S4).

### 3.5. Expression of the TAN-Attracting Chemokines in Basal-Type UBC

**Figure 9:**
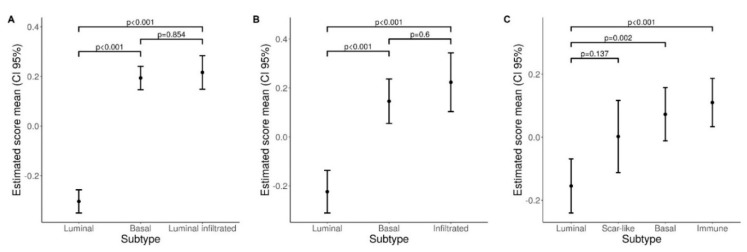
Chemokine signature in datasets. Plots show estimated mean score values and corresponding 95% confidence intervals for Chemokine GSE scores in bladder subtypes (**A** TCGA, **B** GSE32894, **C** GSE124305). Pairwise comparisons p-values were computed from linear models and adjusted for multiple comparisons.

TANs are recruited in the tumor environment mainly via the chemokine (CK) receptors CXCR1 and CXCR2, although many other CKs and coupled receptors could be involved. Of note, similarly to the neutrophil gene signature, we found that a large set of human CKs (Supplementary Figures S5–S10) were significantly over-expressed in basal type, (TCGA, GSE32894 and GSE124305), luminal infiltrated (TCGA) and infiltrated type MIBC (GSE32894), as compared to luminal type (Figure 9). Significantly, among top targets, the IFNγ-inducible CKs (CXCL9, CXCL10 and CXCL11) were included, suggesting increased local availability of IFNγ in these subtypes. Of note, many TAN attracting CKs [55,56], including CXCL1, CXCL2, CX3CL1 and CXCL8 resulted in being highly expressed (Supplementary Figures S8–S10).

**Figure 10:**
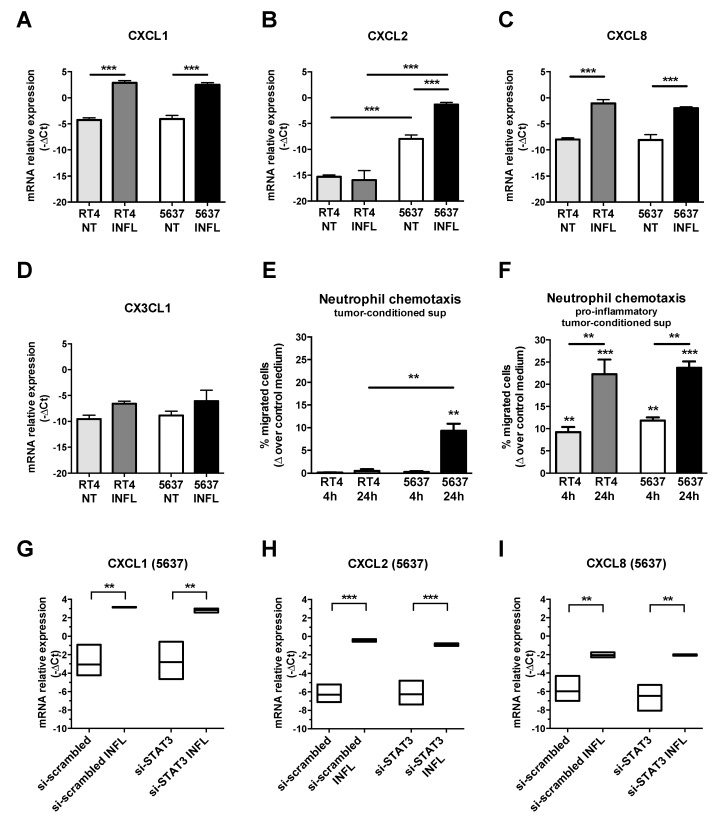
Modulation of TAN-attracting CK in UBC cell line. Column bars (**A**–**D**) showing the mean ± SD of CKs mRNA relative gene expression (-ΔCt, *n* = 3), measured by qRT-PCR in luminal-type RT4 and basal-type 5637 UBC cell lines. After stimulation with a cocktail of proinflammatory cytokines, a significant induction of the CXCL1 and CXCL8 mRNA (**A**,**C**) is observed in both UBC cell lines. CXCL2 is significantly enriched in basal-type 5637 UBC cell line compared to luminal-type RT4 cells both at resting and after stimulation (**B**). Column bars (**E**,**F**) showing neutrophil chemotaxis towards tumor-conditioned supernatants from luminal-type RT4 or basal-type 5637 UBC cell lines untreated (**E**) or stimulated for 4 or 24 h with a pro-inflammatory cytokine cocktail (**F**) (*n* = 3). Chemotaxis is reported as increased percentage of migrated cells (expressed as % of total cell input, mean ± SD) induced by tumor-conditioned supernatants over the control medium. Floating bars (**G**–**I**) showing that CKs’ mRNA is not modulated by siRNA STAT3 in 5637 cell line stimulated with pro-inflammatory cytokine cocktail (mean, range; *n* = 3). A one-way ANOVA test was used for all statistical analysis and pairwise comparisons *p* values were adjusted for multiple comparisons; significant results were showed (* *p* < 0.05, ** *p* < 0.01, *** *p* < 0.001). INFL, with the pro-inflammatory cytokine cocktail (TNF-a, IL6, IL1b); supernatants, sup.

No data are available on the cellular source of these CKs in MIBC. We thus tested the expression of CXCL1, CXCL2, CXCL8 and CX3CL1 CKs by qRT-PCR in luminal type RT4 and basal-type 5637 cell lines (Figure 10A–D). Under resting conditions, both cell lines expressed basal levels of all the tested CKs, with the single exception of CXCL2 which was expressed only by the basal-type 5637 (Figure 10C, *p* < 0.001). Moreover, CXCL1, CXCL2 and CXCL8 were significantly induced by pro-inflammatory stimuli (TNF-a, IL6, IL1b) in both cell lines (Figure 10A–C).

To test whether UBC cells can induce neutrophil migration, a transwell migration assay was performed using peripheral blood neutrophils from healthy donors (Figure 10E–F). Notably, under resting conditions, only supernatants from basal-type 5637 cells induced detectable neutrophil chemotaxis (Figure 10E). However, supernatants obtained from both cell lines after stimulation with the inflammatory cytokine cocktail, were able to induce a significantly increased neutrophil migration as compared to control medium, particularly if derived after 24 h of stimulation (Figure 10F); the latter effect is likely due to the progressive in accumulation of CKs over time.

Based on the TAN enrichment in basal-type UBC showing hyperactivation of STAT3 and FOSL1 expression we tested the modulations of the CK by STAT3 silencing. As we demonstrated, STAT3 silencing in these cell lines showed a marked efficiency by reducing the levels of STAT3 mRNA and protein of about 80% [40]. Of note, si-STAT3 in 5636 UBC line fails to modulate all CKs tested (Figure 10G–I).

## 4. Discussion

Urothelial bladder cancer (UBC) has limited options for systemic treatment. Recent advances in the understanding of their genomic landscape have proposed intrinsic subgroups based on their clinical, histological and biological features [8,9,17]. The two main subgroups are represented by basal and luminal UBC, which likely benefit from completely different treatment approach [10,12,22]. The study proposed here uncover a novel distinct feature of basal type UBC compared to the luminal type, namely a dense infiltration of CD66b^+^ TAN sustained by high expression of TAN-attracting CKs. At the cellular level, pro-inflammatory stimuli induce TAN-attracting CKs, suggesting a local feedback loop involving transformed cells in the organization of the immune microenvironment in basal type UBC.

The most relevant finding of this study is the enrichment of CD66b^+^ TAN in the basal type UBC. This finding was not only suggested by the direct analysis of our MIBC cohort by a specific TAN immunohistochemical marker, but further confirmed by bio-informatic analysis of three distinct UBC datasets showing that a neutrophil signature is enriched in basal-type and luminal-infiltrated UBC. Data from the literature indicate that the immune contexture of basal type UBC is characterized by high amount of tumor infiltrating immune cells [14,15,16]. Basal-type UBC with squamous differentiation contain high density of NK cells, M1 macrophages and memory CD4^+^ T cell showing a Th1-polarization [57]. On the contrary luminal type UBC shows a poor immune infiltrate [14,15], with the exception of a subgroup known as “luminal-infiltrated” [17]. No data are proposed for the TAN immune contexture, even in the more recent consensus classification of UBC. Our bio-informatic analysis indicates that the basal-type and the “luminal infiltrated subtype” are also enriched in chemokines, including those attracting TAN. We subsequently found that TAN infiltration is abundant in MIBC showing hyperactivation of STAT3 and the downstream transcription factor FOSL1. STAT3 is well known as transcription factor relevant in the TAN biology [58]. Evidence suggests that IL-6, a cytokine known for its pro-tumor behavior via JAK2/STAT3 signaling [59], can attract neutrophils within an immunosuppressed microenvironment [60]. Bio-informatic findings from our previous study suggested that also a FOSL1 signature is enriched in basal type UBC and among top regulated genes CXCL8 is included [40]. In this study, we could see a trend of higher density of CD66b^+^ TAN also in FOSL1-expressing MIBC. It has been recently proposed that a FOSL1 signature including a set of TAN-attracting CKs is activated in Basal B type breast cancer cell lines [54]. By analyzing basal and luminal-type BC lines, we found that a set of TAN-attracting CKs including CXCL1, CXCL2 and CXCL8 are potently induced by pro-inflammatory stimuli, with CXCL2 specifically modulated in the basal type MIBC line. Accordingly, we found that only supernatants from basal-type 5637 cells induced detectable neutrophil chemotaxis in resting conditions. It should be noted, however, that pro-inflammatory stimuli induced a significantly increased neutrophil migration in both luminal and basal-type UBC lines.

By blocking STAT3 we were not able to significantly reduce the level of these CKs both resting or after the pro-inflammatory stimuli. This observation is, however, limited to a small set of CKs. All these findings suggest that hyper-activation of STAT3 measured by immunohistochemistry likely represent a surrogate biomarker of basal-type UBC with an active pro-inflammatory environment and high TAN density; however, STAT3 blockade on tumor cells, as recently proposed [40], is likely insufficient to dampen TAN recruitment, since STAT3-independent pathways could also be operative in this system.

An enigmatic finding from this study is represented by the prognostic significance of the TAN density in UBC. A large series of previous studies on the neutrophil to lymphocyte ratio (NLR) in UBC have documented that high NLR predicts worse outcomes in patients who underwent cystectomy [34,35]. Recent studies have investigated the tissue ratio between neutrophils and CD8^+^ T cells in esophageal cancer [61] and in NSCLC [62]. Although in the NSCLC cohort, tissue NLR resulted correlated to response to ICIs, we could not detect any correlations with clinical variables or oncologic outcomes.

On the contrary, very limited data are available on the clinical relevance of TAN. Liu et al. recently showed that elevated CD66b^+^ TAN correlate with an advanced T-stage, a high grade, a worse recurrence-free survival (RFS) within NMIBC subgroup and a worse overall survival (OS) within all UBC cases [36]. Moreover, Zhou et al. have proposed that a high TAN score correlate with a poor OS [37]. It remains unclear how to reconcile our finding (predictive of better prognosis) with these and other studies [32]. Technical differences in definition of the region of interest (whole slide vs. hot spot or random sampling) and type of measurement (semiquantitative vs. absolute counting) could account. Moreover, recent advances in the characterization of TAN populations have identified TAN with anti-tumor function [63], particularly in the early stages cancer [64]. It should be noted that in UBC, a relevant anti-tumor activity of TAN has been proposed in response to BCG treatment [65], also supported by a recent work in a murine model [66]. Moreover, a high urinary level of TRAIL (tumor necrosis factor-related apoptosis-inducing ligand), a key factor for neutrophil antitumor activity [67], predicts favorable response to BCG therapy [68]. Finally, a high urinary level of IL-8, a well-known chemokine attracting neutrophil [69], is correlated to better disease free survival after BCG therapy [70]. Finally, genomic and proteomic approaches have proposed the existence of numerous TAN sub-populations [71], a level of heterogeneity that could not be resolved by a single marker.

From a statistical perspective, TAN density in our study although associated with a good outcome, also correlate with the density of CD3^+^ T cells, suggesting a potential synergy of the two populations. However, compared to the linear predicting model followed by the CD3 density, the CD66 variable showed a weaker predicting power based testified by its non-linear behavior. These findings suggest that the identification of additional TAN biomarkers might significantly improve the level of immune contexture taxonomy.

Basal-type and luminal-infiltrated subtypes are ‘hot tumors’ heavily infiltrated by T cells [57]. According to different consensus clustering, “luminal-infiltrated” subtypes include tumors with high CD8^+^ T cell gene signature expression, high TMB and neo-antigens and a wild-type TP53 signature that make them prone to better responses to ICIs. Basal-type MIBC, display a high PD-L1 expression, as also supported by the high level of IFN-γ inducible CKs CXCL9-11 (this study). They show a high frequency of TP53 mutations and a defective DNA damage repair system that make them more susceptible to chemotherapy with DNA-damaging agents [5]. The apoptotic effect of NeoAdjuvant Chemotherapy (NAC) may promote local chemokine release and subsequent immune cell recruitment to the tumor site facilitating the response to a second-line immune therapy [45]. Our findings suggest that as a whole these “hot” UBC could also be heavily infiltrated by TAN.

## 5. Conclusions

In conclusion, the analysis of TAN in prospective large-cohorts of molecularly-define BC is highly legitimated. Integration with surrogate biomarkers of functional activation (i.e., TRAIL or TNF expression) might offer additional criteria for patient stratification for appropriate co-targeting of TAN.

## Figures and Tables

**Table 1:** Demographic, histological, and clinical data of the MIBC patient cohort. CIS, carcinoma in situ; SD, standard deviation; IQR, interquartile range; NA, not available.

Total	N (%)	CIS Concomitant	N (%)
	84 (100)		
		Yes	50 (60)
Gender		No	34 (40)
Female	16 (19)	Global Stage	
Male	68 (81)	II	22 (26)
Age (years)		III	33 (39)
Mean (SD)	70.9 (10.44)	IV	29 (35)
Median (IQR)	72 (14)	Subtype	
Stage pT		Luminal	41 (49)
pT2	25 (30)	Basal	20 (24)
pT3	41 (49)	Non-type	23 (27)
pT4	18 (21)	pSTAT3	
Stage pN		Score 0–1	43 (51)
pN0	57 (68)	Score 2–3	41 (49)
pN1	16 (19)	FOSL1	
pN2	10 (12)	Score 0–1	19 (23)
pN3	1 (1)	Score 2–3 NA	22 (26) 43 (51)

**Table 2:** Univariate analysis of clinical and pathological findings related to overall survival and progression free survival. tNLR, tissue NLR.

Clinicopathological Variables	Overall Survival		Progression Free Survival	
	H.R. (CI 95%)	*p*	H.R. (CI 95%)	*p*
Age (years)	1.04 (1.01–1.08)	0.024	0.99 (0.96–1.02)	0.65
Gender (F vs. M)	2.93 (1.45–5.94)	0.003	1.94 (0.94–4.00)	0.072
pT (pT3-4 vs. pT2)	4.21 (1.63–10.85)	0.003	3.54 (1.47–8.55)	0.005
pN (pN+ vs. pN0)	2.22 (1.16–4.23)	0.016	2.25 (1.17–4.33)	0.015
CIS (yes vs. no)	0.63 (0.33–1.19)	0.155	0.86 (0.45–1.65)	0.657
Overall Stage (SIII-IV vs. SII)	5.07 (1.78–14.41)	0.002	3.94 (1.52–10.21)	0.005
Subtype (basal vs. luminal)	1.3 (0.59–2.87)	0.520	0.70 (0.32–1.55)	0.383
Subtype (Non-type vs. luminal)	1.66 (0.78–3.53)	0.191	0.73 (0.33–1.60)	0.431
pSTAT3 (High vs. Low)	1.19 (0.63–2.26)	0.595	0.69 (0.36–1.32)	0.265
CD66b (Low vs. High)	2.53 (1.30–4.92)	0.006	2.68 (1.37–5.24)	0.004
CD3 (Low vs. High)	2.62 (1.35–5.08)	0.004	1.69 (0.88–3.22)	0.11
CD3CD66b Immunoscore (LoLo vs. HiHi)	5.27 (2.12–13.11)	<0.001	3.95 (1.53–10.16)	0.004
CD3CD66b Immunoscore (LoHi vs. HiHi)	2.26 (0.79–6.49)	0.13	2.15 (0.75–6.14)	0.155
CD3CD66b Immunoscore (HiLo vs. HiHi)	2.18 (0.79–6.04)	0.133	3.43 (1.32–8.89)	0.011
tNLR (CD66b^+^/CD3^+^)	1.04 (0.75–1.44)	0.828	1.07 (0.75–1.53)	0.699

**Table 3:** Association analysis between immune cell infiltration and clinic-pathological data. tNLR, tissue NLR; CIS, carcinoma in situ; *p* value estimated by # Kruskal-Wallis test or * Mann-Whitney test.

Clinicopathological Variables		CD66b^+^ TAN Density		CD3^+^ T Cells Density		tNLR (CD66b^+^/CD3^+^)	
	N° (%)	Median	*p*	Median	*p*	Median	*p*
Total	84 (100)	78		396			
Gender			0.76 *		0.47 *		0.90 *
Female	16 (19)	51		328		0.186	
Male	68 (81)	86		399		0.216	
Age			0.80 *		0.26 *		0.55 *
< median	39 (46)	83		327		0.186	
≥ median	45 (54)	73		415		0.224	
pT category			0.11 *		0.003 *		0.35 *
pT2	25 (30)	106		544		0.136	
pT3-pT4	59 (70)	60		304		0.292	
pN category			0.10 *		0.19 *		0.50 *
pN+	27 (32)	106		403		0.154	
pN0	57 (68)	48		357		0.234	
Overall Stage			0.14 *		0.001 *		0.32 *
II	22 (26)	123		587		0.149	
III-IV	62 (74)	56		312		0.263	
CIS			0.46 *		0.65 *		0.90 *
No	34 (40)	107		381		0.183	
Yes	50 (60)	68		402		0.239	
pSTAT3			0.048 *		0.43 *		0.28 *
Low	43 (51)	51		357		0.176	
High	41 (49)	126		400		0.243	
Subtype			0.007#		0.75 ^#^		0.06 ^#^
Luminal	41 (49)	51		401		0.136	
Basal	20 (24)	178		398		0.359	
Non-Type	23 (27)	60		366		0.304	
FOSL1			0.054 *		0.227 *		0.55 *
Low	19 (22)	58		418		0.291	
High	22 (26)	155		398		0.281	

**Table 4:** Multivariable survival analysis.

Overall Survival		
	H.R. (CI 95%)	*p*
Age (year)	1.041 (1.006–1.077)	0.019
Gender (F)	2.83 (1.33–5.99)	0.01
pT (pT3-4)	3.67 (1.28–10.51)	0.016
CD3CD66b Immunoscore (LoLo)	6.67 (2.43–18.25)	<0.001
CD3CD66b Immunoscore (LoHi)	4.20 (1.35–13.11)	0.013
CD3CD66b Immunoscore (HiLo)	1.59 (0.55–4.61)	0.40
**Progression Free Survival**		
pT (pT3-4)	3.58 (1.44–8.93)	0.006
CD3CD66b Immunoscore (LoLo)	3.55 (1.35–9.34)	0.01
CD3CD66b Immunoscore (LoHi)	2.66 (0.91–7.84)	0.08
CD3CD66b Immunoscore (HiLo)	3.69 (1.41–9.64)	0.008

Reference categories: Gender, M; pT, pT2; CD3CD66b Immunoscore, HiHi.

## Supplementary Materials

The following are available online at https://www.mdpi.com/2073-4409/9/2/291/s1, Figure S1: HR estimate and CI95% for OS and PFS; Figure S2: hierarchical clustering and gene expression heatmaps of the neutrophil gene signature (TCGA dataset); Figure S3: hierarchical clustering and gene expression heatmaps of the neutrophil gene signature (GSE32894 dataset); Figure S4: hierarchical clustering and gene expression heatmaps of the neutrophil gene signature (GSE124305 dataset); Figure S5: hierarchical clustering and gene expression heatmaps of the chemokine gene signature (TCGA dataset);Figure S6: hierarchical clustering and gene expression heatmaps of the chemokine gene signature (GSE32894 dataset); Figure S7: hierarchical clustering and gene expression heatmaps of the chemokine gene signature (GSE124305 dataset); Figure S8: Waterfall Plot comparing Basal/Luminal Infiltrated vs. Luminal subtype for every Chemokine (TCGA dataset); Figure S9: Waterfall Plot comparing Basal/Infiltrated vs. Luminal subtype for every Chemokine (GSE32894 dataset); Figure S10: Waterfall Plot comparing Basal/Immune vs. Luminal subtype for every Chemokine (GSE124305 dataset); Table S1: fully details (dataset) of clinical and pathology data of the retrospective MIBC cohort. Table S2: details of reagents for the Gene Expression Assay on UBC lines. Table S3: PMN and Chemokine signatures used for Analysis of the TCGA, GSE32894 and GSE124305 Datasets. Table S4: summary statistics of CD3 and CD66b as continuous variable.
