# Supplementary material for: Tumor Infiltrating Neutrophils Are Enriched in Basal-Type Urothelial Bladder Cancer
Source: Cells. 2020 Jan 25;9(2):291. doi: 10.3390/cells9020291 (PMC7072276; doi:10.3390/cells9020291)
Supplement: Supplementary file 1 [file cells-09-00291-s001.zip › SM/Mandelli et al.SupplementaryFiguresR1 .pdf]

# Tumor infiltrating neutrophils are enriched in basal-type urothelial bladder cancer

**Giulio Eugenio Mandelli [1, †], Francesco Missale [1, 2,3, †], Debora Bresciani [1], Luisa Benerini Gatta [1, 4], Patrizia Scapini [5], Elena Caveggion [5], Elisa Roca [4, 6], Mattia Bugatti [1, 6], Matilde Monti [1], Luca Cristinelli [4, 6], Sandra Belotti [4, 6], Claudio Simeone [4, 6], Stefano Calza [1], Laura Melocchi [7] and William Vermi [1, 6, 8 *]**

[1] Department of Molecular and Translational Medicine, School of Medicine, University of Brescia, 25100 Brescia, Italy
[2] IRCCS Ospedale Policlinico San Martino, 16121 Genova, Italy
[3] Department of Otorhinolaryngology, Head and Neck Surgery—University of Genoa, 16121 Genova, Italy
[4] Department of Medical and Surgical Specialties, Radiological Sciences, and Public Health, University of Brescia, 25100 Brescia, Italy
[5] Section of General Pathology, Department of Medicine, University of Verona, Verona, Italy
[6] ASST Spedali Civili di Brescia, 25100 Brescia, Italy
[7] Department of Pathology, Fondazione Poliambulanza, 25100 Brescia, Italy
[8] Department of Pathology and Immunology, Washington University School of Medicine, St. Louis, MO 63130, USA

## Supplementary Figures

**Supplementary Figure 1** shows HR estimate and 95% CI for OS and PFS

**Supplementary Figure 2** shows hierarchical clustering and gene expression heatmaps of the neutrophil gene signature (TCGA dataset)

**Supplementary Figure 3** shows hierarchical clustering and gene expression heatmaps of the neutrophil gene signature (GSE32894 dataset)

**Supplementary Figure 4** shows hierarchical clustering and gene expression heatmaps of the neutrophil gene signature (GSE124305 dataset)

**Supplementary Figure 5** shows hierarchical clustering and gene expression heatmaps of the chemokine gene signature (TCGA dataset)

**Supplementary Figure 6** shows hierarchical clustering and gene expression heatmaps of the chemokine gene signature (GSE32894 dataset)

**Supplementary Figure 7** shows hierarchical clustering and gene expression heatmaps of the chemokine gene signature (GSE124305 dataset)

**Supplementary Figure 8** display a Waterfall Plot comparing Basal/Luminal Infiltrated vs Luminal subtype for every Chemokine (TCGA dataset)

**Supplementary Figure 9** display a Waterfall Plot comparing Basal/Infiltrated vs Luminal subtype for every Chemokine (GSE32894 dataset)

**Supplementary Figure 10** display a Waterfall Plot comparing Basal/Immune vs Luminal subtype for every Chemokine (GSE124305 dataset)

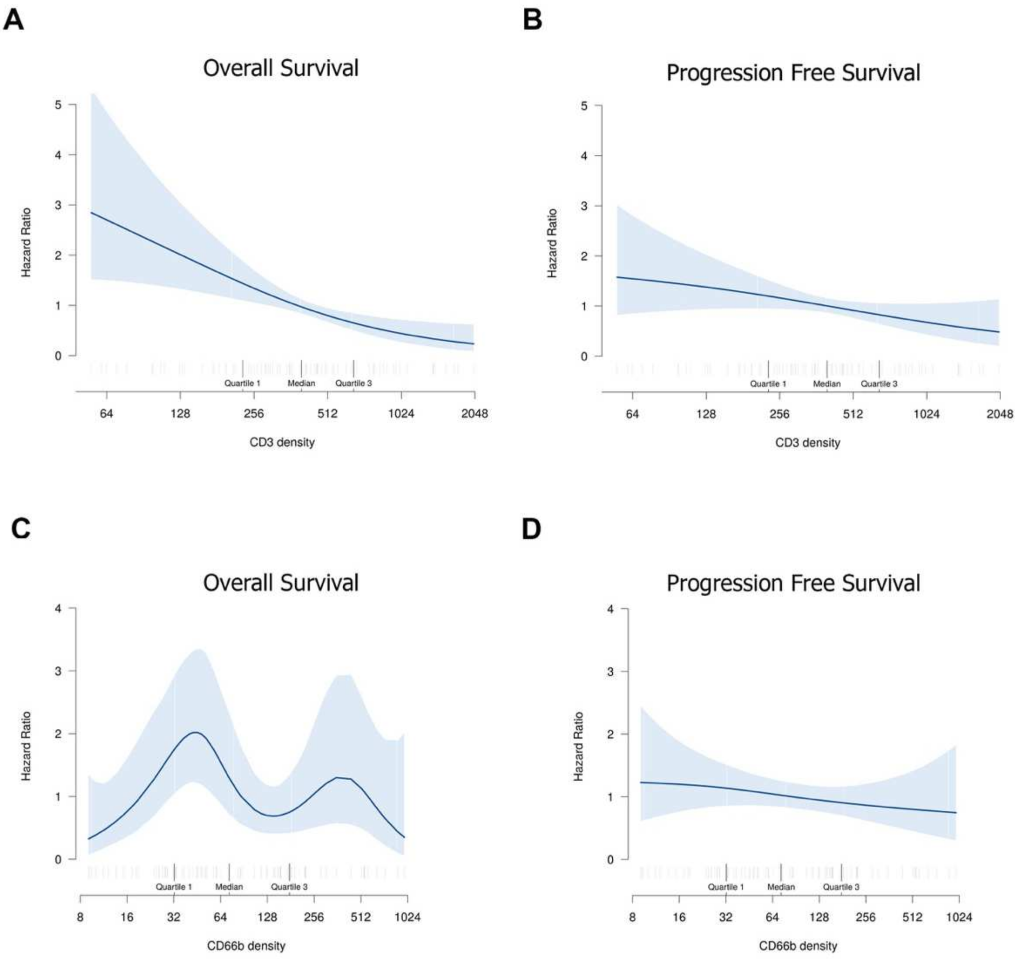


**Supplementary Figure 1.** HR estimate (dark line) and 95% CI (dashed area) for OS and PFS according to CD3⁺ T cell density (**A** and **B**) and CD66b⁺ TAN density (**B** and **C**), modelled by penalized splines. Reference for HR estimate: mean risk of the whole cohort.

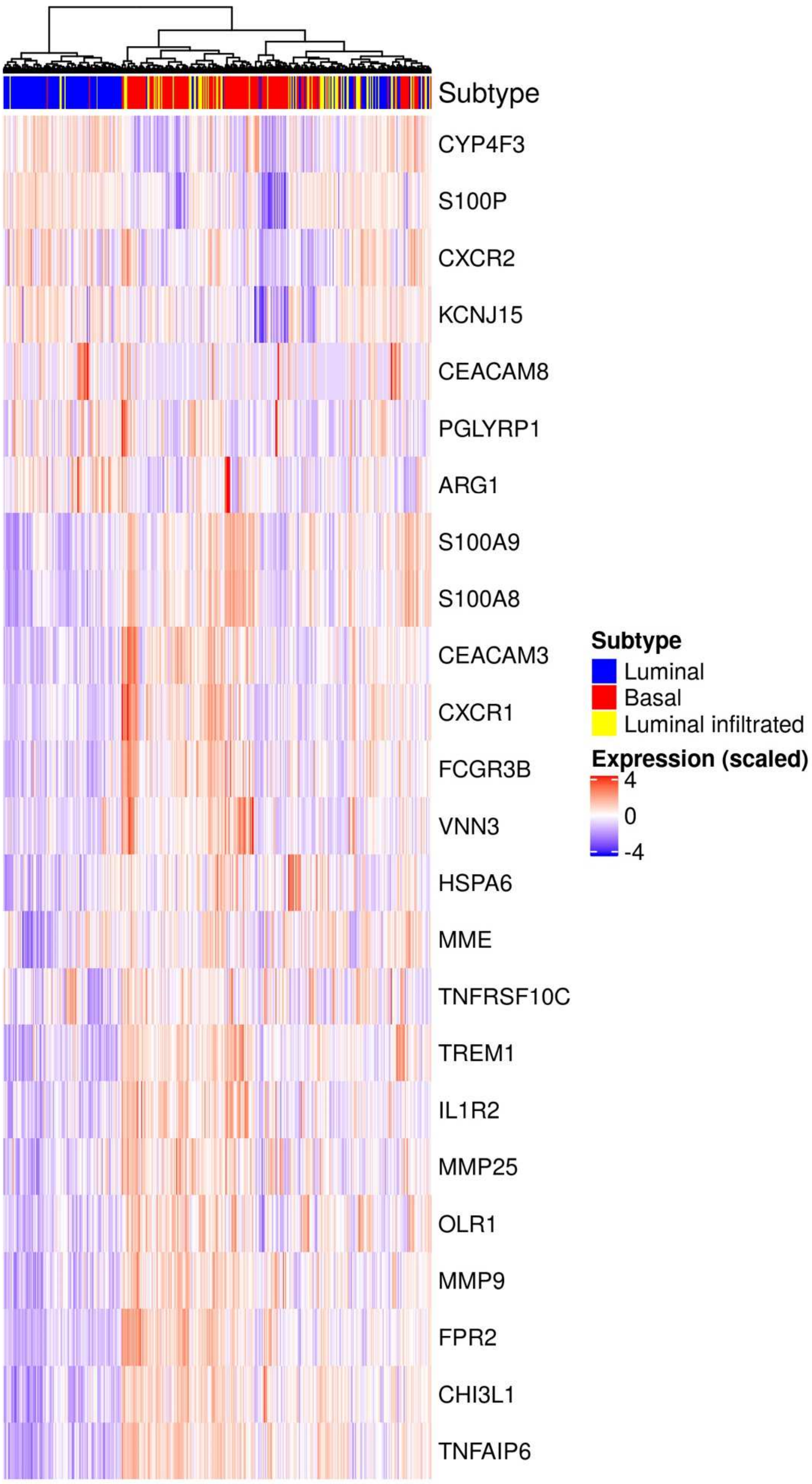


**Supplementary Figure 2.** Hierarchical clustering with gene expression heatmaps for TCGA dataset considering the neutrophil gene signature. Gene-level dendrograms were computed using Euclidean distance while sample-level clustering was based on random forest derived proximity matrix. Both clustering used Ward algorithm. Gene expression values were scaled and mean centered.

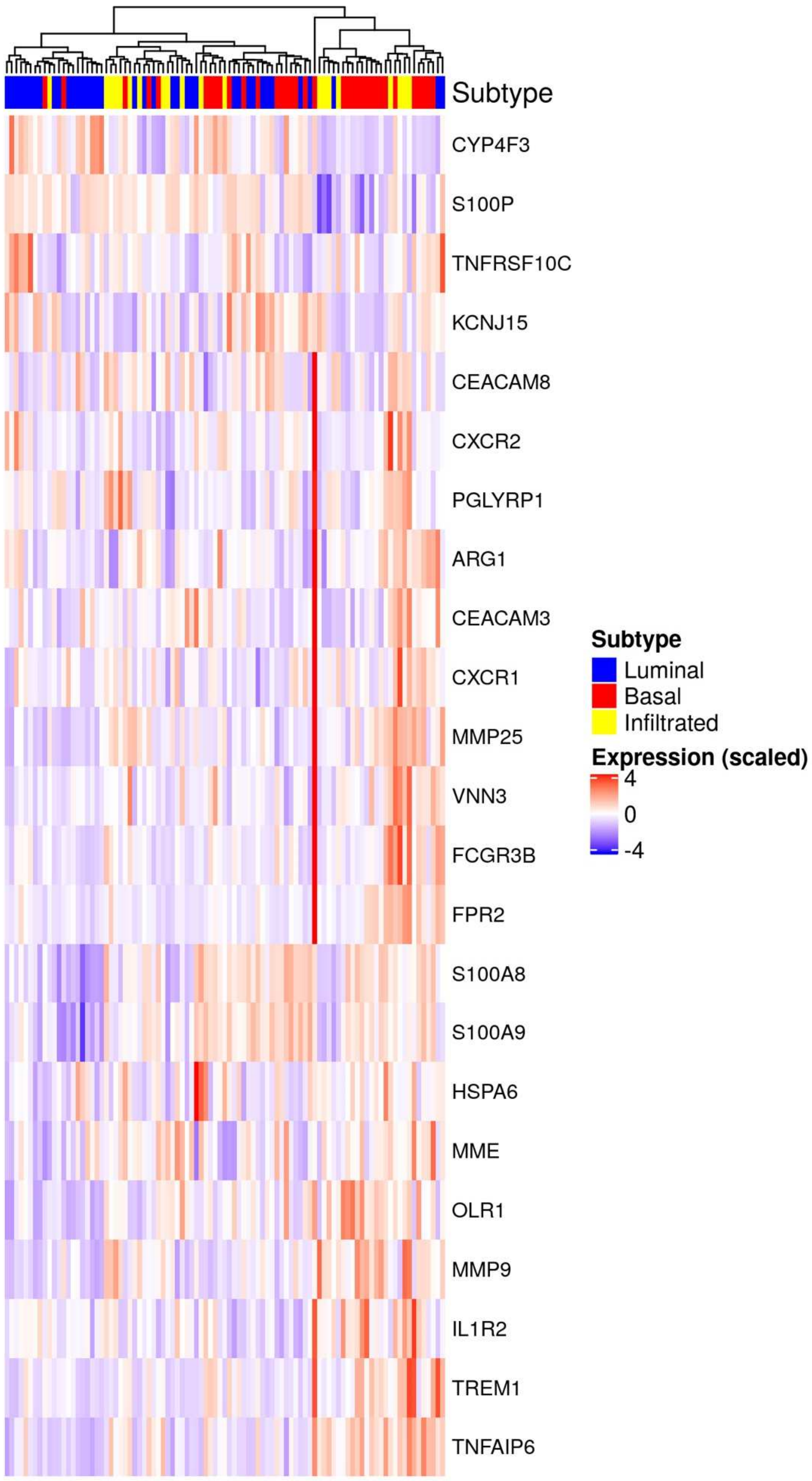


**Supplementary Figure 3.** Hierarchical clustering with gene expression heatmaps for GSE32894 dataset considering the neutrophil gene signature. Gene-level dendrograms were computed using Euclidean distance while sample-level clustering was based on random forest derived proximity matrix. Both clustering used Ward algorithm. Gene expression values were scaled and mean centered.

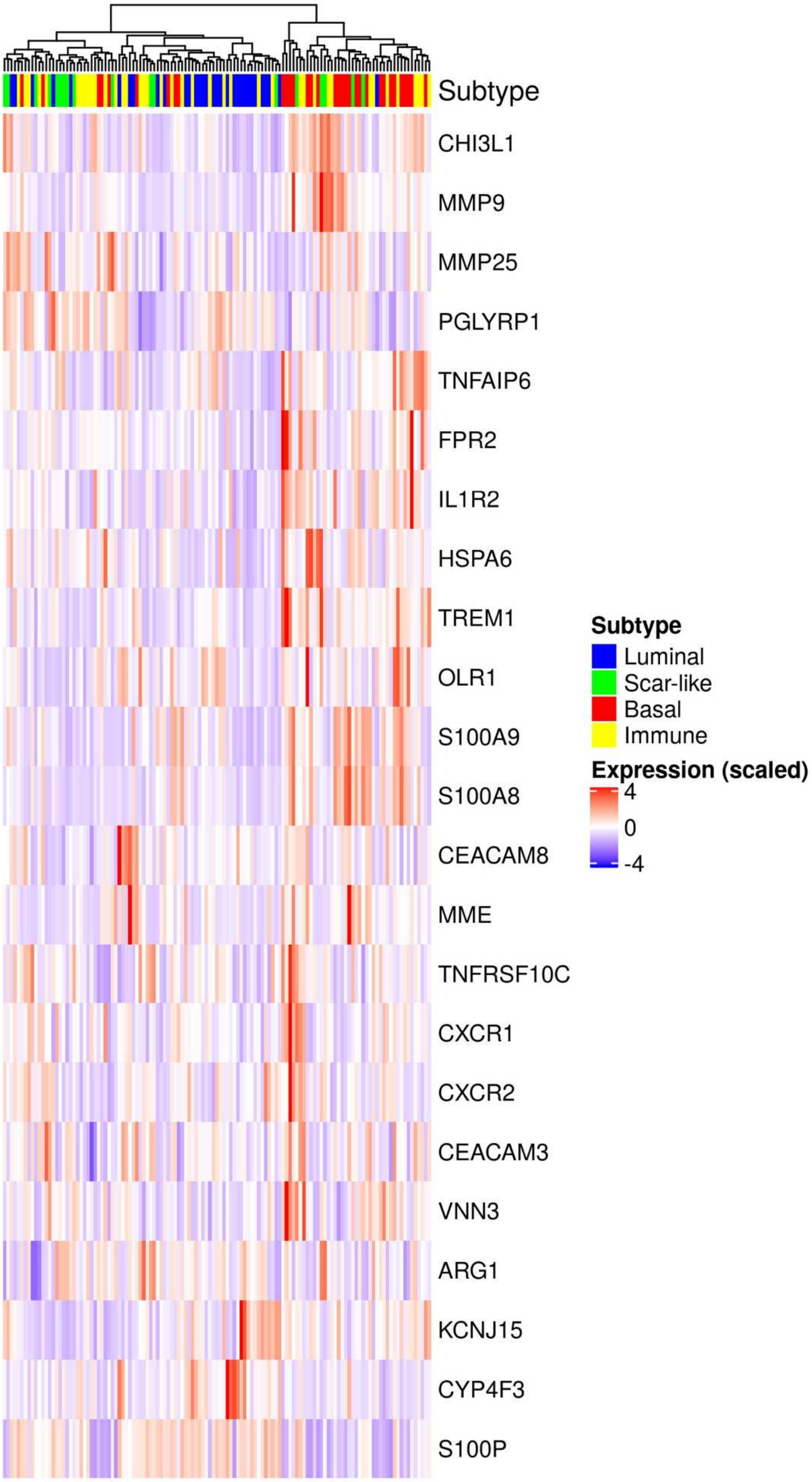


**Supplementary Figure 4.** Hierarchical clustering with gene expression heatmaps for GSE124305 dataset considering the neutrophil gene signature. Gene-level dendrograms were computed using Euclidean distance while sample-level clustering was based on random forest derived proximity matrix. Both clustering used Ward algorithm. Gene expression values were scaled and mean centered.

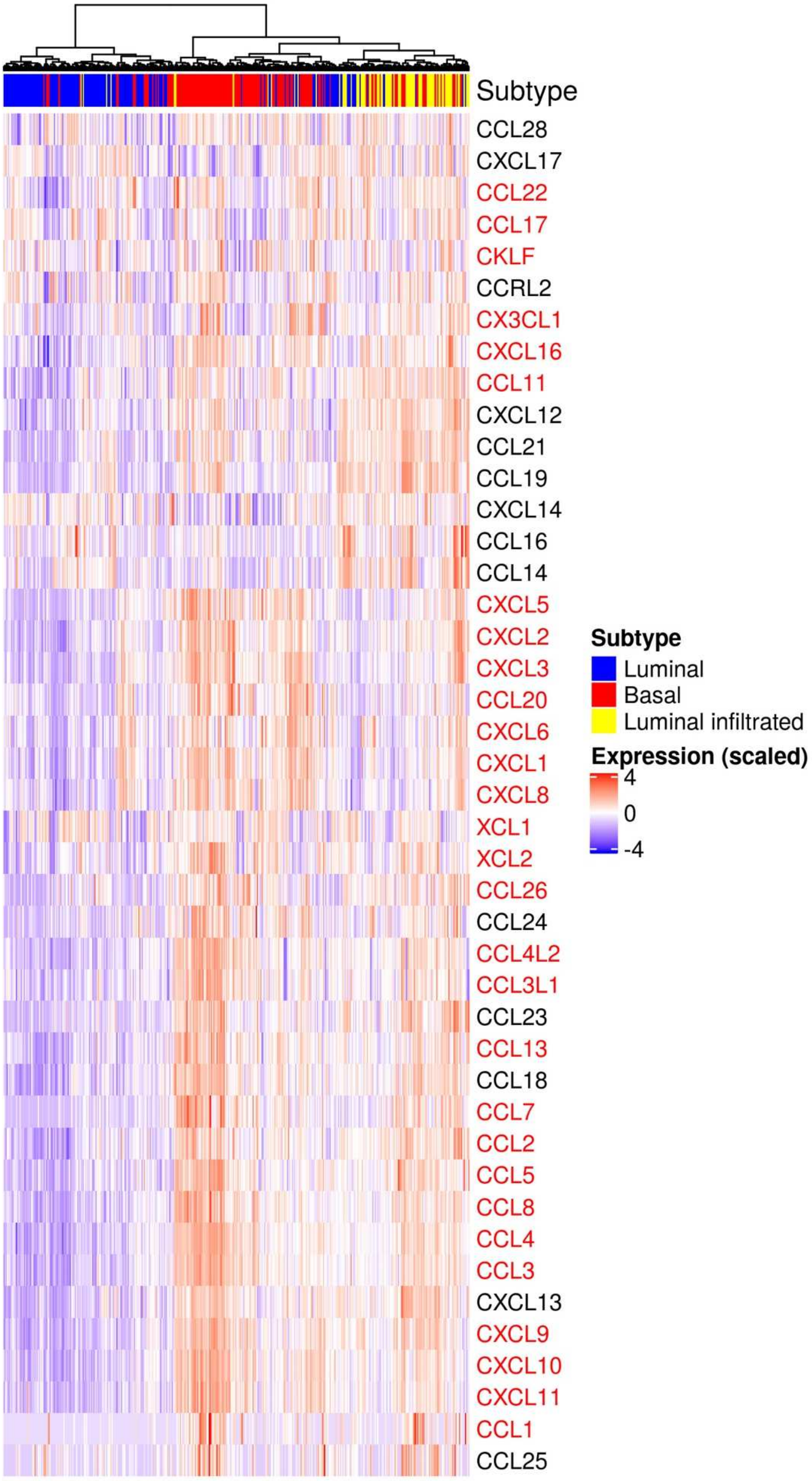


**Supplementary Figure 5.** Hierarchical clustering with gene expression heatmaps for TCGA dataset considering the chemokine gene signature. Both gene-level and sample-level dendrograms were computed using Euclidean distance and Ward algorithm. Gene expression values were scaled and mean centered. Chemokines with pro-inflammatory functions are labeled in red.

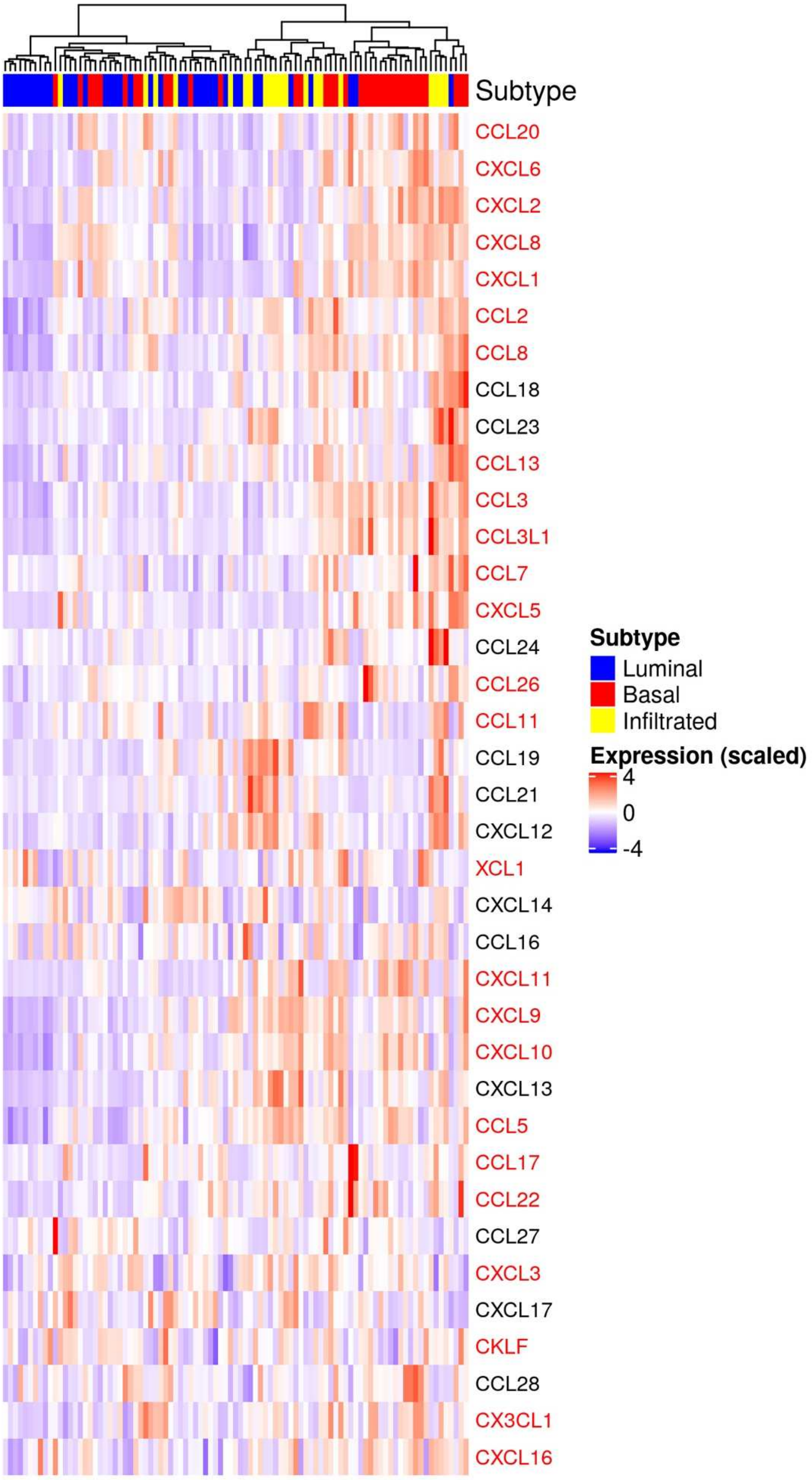


**Supplementary Figure 6.** Hierarchical clustering with gene expression heatmaps for GSE32894 dataset considering the chemokine gene signature. Both gene-level and sample-level dendrograms were computed using Euclidean distance and Ward algorithm. Gene expression values were scaled and mean centered. Chemokines with pro-inflammatory functions are labeled in red.

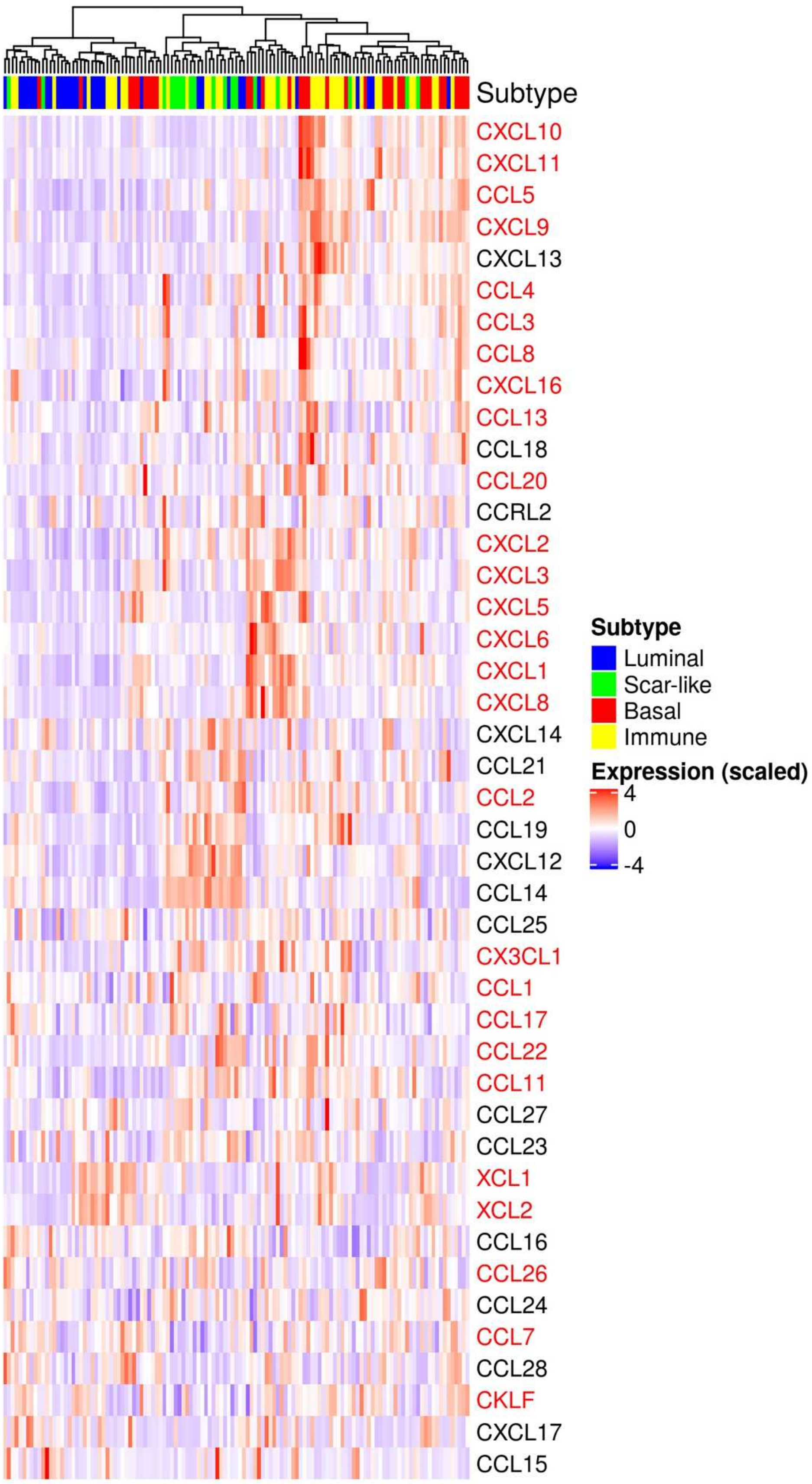


**Supplementary Figure 7.** Hierarchical clustering with gene expression heatmaps for GSE124305 dataset considering the chemokine gene signature. Both gene-level and sample-level dendrograms were computed using Euclidean distance and Ward algorithm. Gene expression values were scaled and mean centered. Chemokines with pro-inflammatory functions are labeled in red.

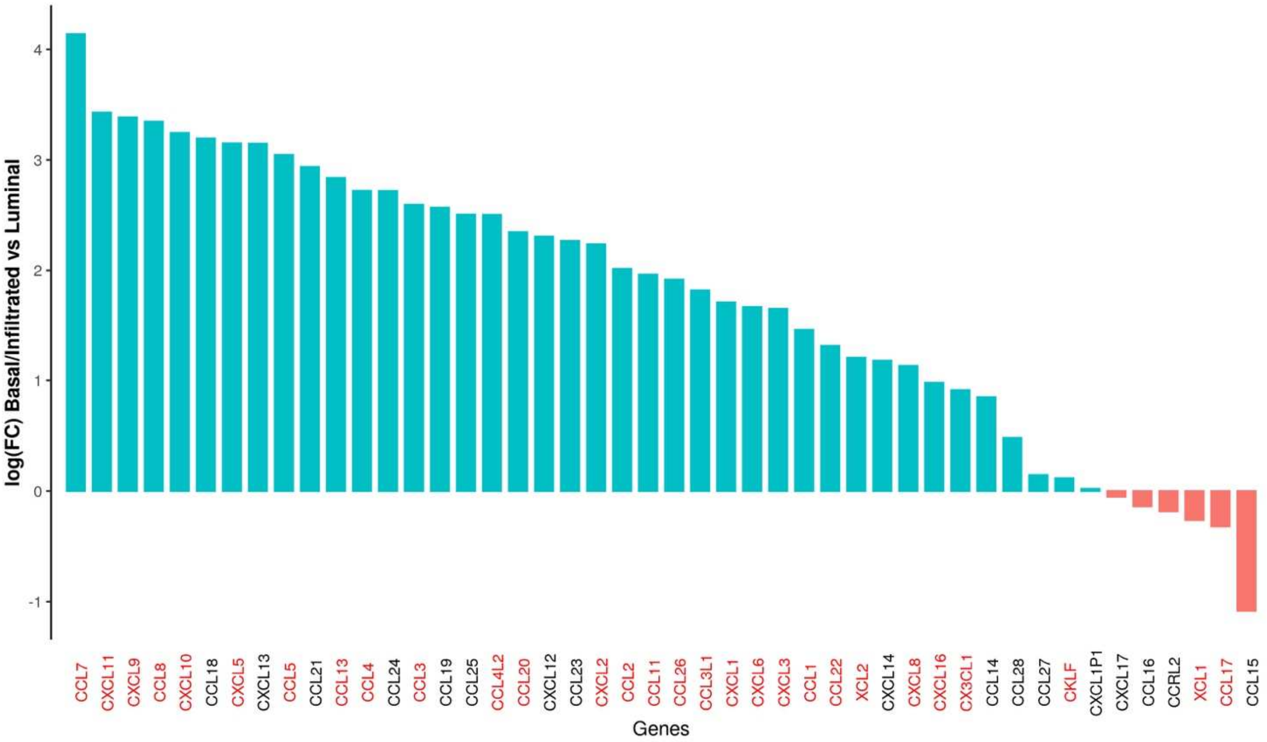


**Supplementary Figure 8.** Waterfall plots showing Fold Change values (on log2 scale) comparing Basal/Infiltrated vs Luminal subtype for every Chemokine of the signature in TCGA dataset. Chemokines with pro-inflammatory functions are labeled in red.

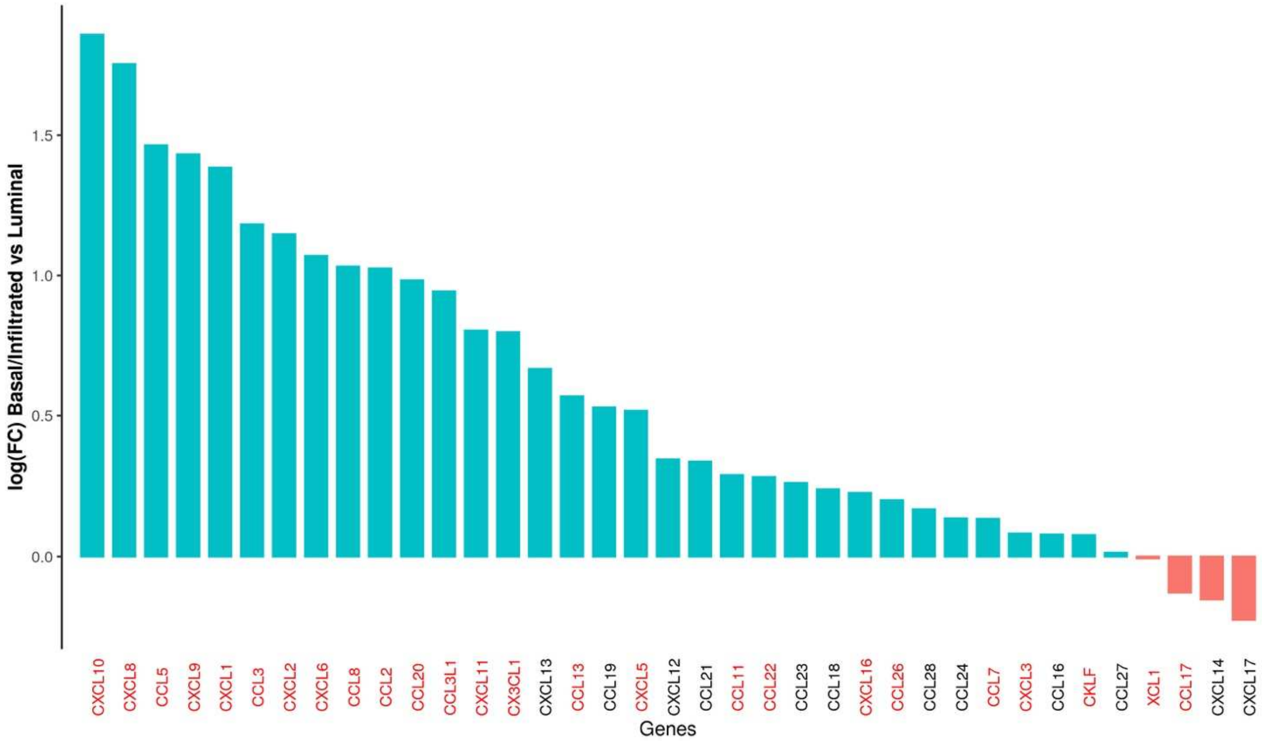


**Supplementary Figure 9.** Waterfall plots showing Fold Change values (on log2 scale) comparing Basal/Infiltrated vs Luminal subtype for every Chemokine of the signature in GSE32894 dataset. Chemokines with pro-inflammatory functions are labeled in red.

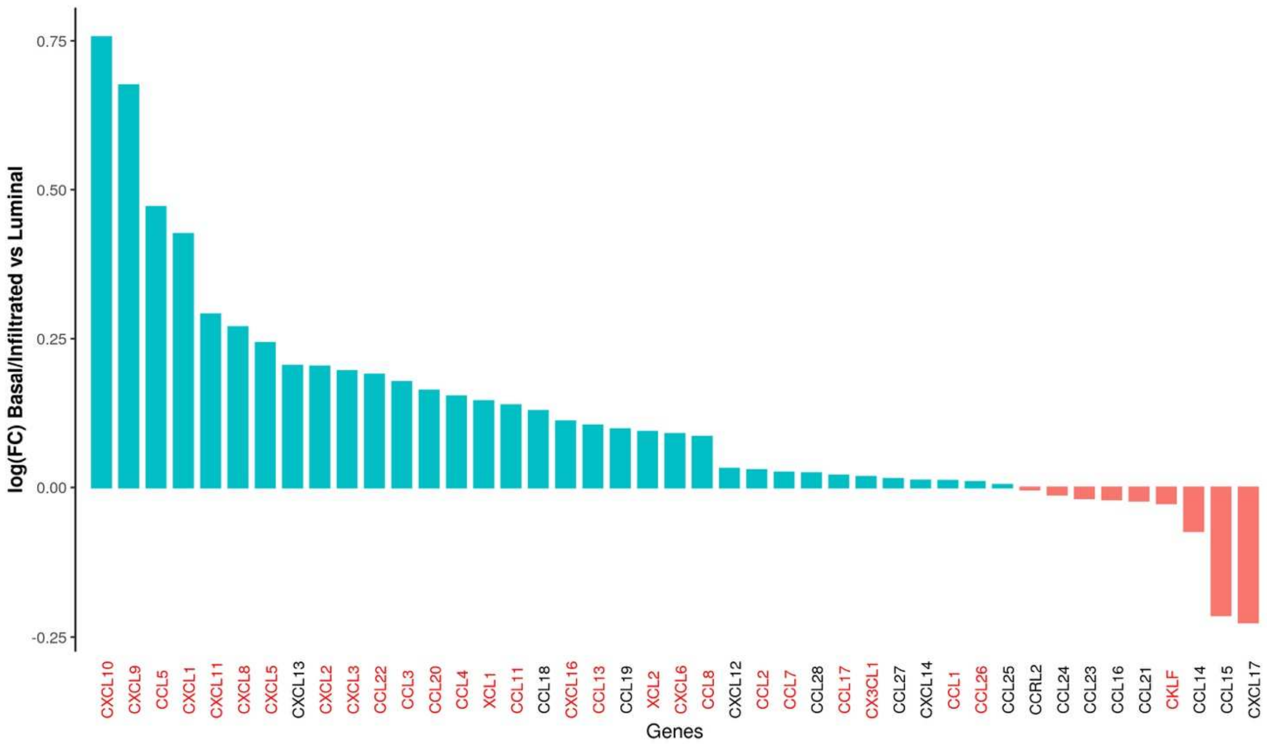


**Supplementary Figure 10.** Waterfall plots showing Fold Change values (on log2 scale) comparing Basal/Infiltrated vs Luminal subtype for every Chemokine of the signature in GSE124305 dataset. Chemokines with pro-inflammatory functions are labeled in red.
