# Supplementary material for: Tumor Infiltrating Neutrophils Are Enriched in Basal-Type Urothelial Bladder Cancer
Source: Cells. 2020 Jan 25;9(2):291. doi: 10.3390/cells9020291 (PMC7072276; doi:10.3390/cells9020291)
Supplement: Supplementary file 1 [file cells-09-00291-s001.zip › SM/Mandelli et al.SupplementaryTablesR1.pdf]

Tumor infiltrating neutrophils are enriched in basal-type urothelial bladder cancer

Giulio Eugenio Mandelli [1,†], Francesco Missale [1,2,3,†], Debora Bresciani [1], Luisa Benerini Gatta [1,4], Patrizia Scapini [5], Elena Caveggion [5], Elisa Roca [4,6], Mattia Bugatti [1,6], Matilde Monti [1], Luca Cristinelli [4,6], Sandra Belotti [4,6], Claudio Simeone [4,6], Stefano Calza [1], Laura Melocchi [7] and William Vermi [1,6,8*]

[1] Department of Molecular and Translational Medicine, School of Medicine, University of Brescia, 25100 Brescia, Italy
[2] IRCCS Ospedale Policlinico San Martino, 16121 Genova, Italy
[3] Department of Otorhinolaryngology, Head and Neck Surgery—University of Genoa, 16121 Genova, Italy
[4] Department of Medical and Surgical Specialties, Radiological Sciences, and Public Health, University of Brescia, 25100 Brescia, Italy
[5] Section of General Pathology, Department of Medicine, University of Verona, Verona, Italy
[6] ASST Spedali Civili di Brescia, 25100 Brescia, Italy
[7] Department of Pathology, Fondazione Poliambulanza, 25100 Brescia, Italy
[8] Department of Pathology and Immunology, Washington University School of Medicine, St. Louis, MO 63130, USA
* Correspondence: william.vermi@unibs.it; Tel.:+39-030-399-8425
† These authors equally contributed to the work.

Supplementary Tables

Supplementary Table S1 reports the details of clinical and pathology data of the retrospective MIBC cohort.

Supplementary Table S2 reports the details of reagents for the Gene Expression Assay on UBC lines.

Supplementary Table S3: reports the neutrophil and chemokine signatures used for Analysis of the TCGA, GSE32894 and GSE124305 Datasets.

Supplementary Table S4 reports summary statistics of CD3 and CD66b as continuous variable, p values by Shapiro-Wilk normality test.

Supplementary Table S1. Details of clinical data of the retrospective MIBC cohort.

ID	Density CD66B (cell/mm^2)	Density CD3 (cell/mm^2)	Immunoscore	Age	Gender	pT	pN	CIS	STAGE	PFS cens (1=yes 0=no)	PFS time (months)	Status: (1=Died 0=Alive)	OS time	pSTAT3	pSTAT3 HiLo	Subtype	FOSL1
1	148,9	1376,0	3_HiHi	51	M	T2a	N0	0	2	0	108	0	108	2	Hi	Luminal	
2	732,3	248,9	1_3LoHi	68	M	T2b	N0	0	2	0	92	1	92	3	Hi	Basal	3
3	282,5	788,4	3_HiHi	82	M	T4	N0	0	3	0	89	1	89	3	Hi	Basal	3
4	975,6	2020,8	3_HiHi	73	M	T3b	N0	0	3	1	84	0	108	3	Hi	Basal	2
5	86,2	788,2	3_HiHi	68	M	T3b	N1	1	4	0	108	0	108	1	Lo	Luminal	
6	60,2	288,8	2_3HiLo	57	M	T2b	N0	1	2	0	96	0	96	2	Hi	Non-type	
7	225,4	1385,3	3_HiHi	52	M	T2b	N0	0	2	0	101	0	101	1	Lo	Basal	3
8	32,7	1557,8	2_3HiLo	78	M	T3a	N0	1	3	0	96	0	96	3	Hi	Non-type	
9	143,5	400,2	3_HiHi	73	M	T3a	N0	1	3	0	96	0	96	3	Hi	basal	2
10	105,9	60,9	1_3LoHi	54	F	T2b	N0	1	2	0	84	0	84	2	Hi	luminal	1
11	1048,9	467,9	3_HiHi	78	M	T3a	N0	1	3	0	84	0	84	2	Hi	Non-type	
12	156,3	536,7	3_HiHi	70	F	T2b	N0	1	2	0	84	0	84	2	Hi	Luminal	1
13	139,9	327,1	3_HiHi	61	M	T2b	N0	1	2	0	72	0	72	3	Hi	Basal	3
14	155,2	638,2	3_HiHi	69	M	T2	N0	1	2	0	72	0	72	2	Hi	Basal	3
15	31,7	400,6	2_3HiLo	71	M	T2b	N0	0	2	0	54	0	54	1	Lo	Luminal	2
16	86,0	1745,1	3_HiHi	76	M	T3	N0	0	3	0	60	0	60	1	Lo	Non-type	
17	17,3	453,1	2_3HiLo	69	M	T3a	N0	0	3	0	36	0	36	0	Lo	Non-type	
18	117,2	348,8	3_HiHi	50	M	T4a	N0	1	3	0	36	0	36	1	Lo	Non-type	1
19	356,0	263,5	1_3LoHi	65	M	T4	N0	0	3	1	3	1	14	3	Hi	Basal	3
20	12,1	27,3	0_LoLo	73	M	T3b	N2	1	4	1	1	1	50	1	Lo	Non-type	
21	172,6	892,8	3_HiHi	90	F	T2b	N0	0	2	0	65	1	65	3	Hi	Basal	3
22	11,2	214,4	0_LoLo	57	M	T3a	N0	0	3	1	20	1	25	2	Hi	Non-type	
23	9,2	671,7	2_3HiLo	75	M	T3a	N1	1	4	1	18	0	132	1	Lo	Luminal	0
24	47,9	461,5	2_3HiLo	80	F	T2b	N1	1	4	1	2	1	4	2	Hi	Luminal	0
25	10,1	283,4	0_LoLo	60	M	T3a	N0	1	3	1	9	1	10	2	Hi	Non-type	
26	25,6	564,3	2_3HiLo	65	M	T2b	N0	1	2	0	85	1	85	1	Lo	Luminal	
27	127,4	436,0	3_HiHi	76	M	T3a	N1	1	4	1	51	1	92	1	Lo	Luminal	1
28	223,7	357,5	3_HiHi	65	M	T2a	N0	1	2	1	38	0	91	1	Lo	Luminal	
29	18,3	98,2	0_LoLo	58	M	T3a	N0	0	3	1	5	1	7	3	Hi	Basal	3
30	60,0	464,3	2_3HiLo	86	F	T3b	N0	0	3	1	16	1	27	3	Hi	Basal	0
31	565,8	418,2	3_HiHi	62	M	T4	N0	1	3	1	9	0	72	1	Lo	Luminal	0
32	48,7	484,2	2_3HiLo	70	M	T2b	N0	1	2	1	4	0	68	1	Lo	Luminal	

33	24,8	397,2	2_3HiLo	68	M	T3a	N2	0	4	1	1	1	11	2	Hi	Non-type	
34	15,2	196,8	0_LoLo	59	M	T2a	N0	0	2	1	4	0	47	1	Lo	Luminal	1
35	307,5	462,8	3_HiHi	66	M	T3a	N0	0	3	1	4	1	11	3	Hi	Basal	3
36	71,7	975,7	2_3HiLo	61	M	T2b	N0	1	2	1	24	1	46	3	Hi	Non-type	
37	154,9	507,7	3_HiHi	75	M	T3b	N2	1	4	1	19	1	33	1	Lo	Luminal	
38	33,3	500,6	2_3HiLo	81	F	T3a	N3	1	4	1	10	1	35	1	Lo	Luminal	0
39	32,8	63,8	0_LoLo	74	M	T3a	N0	0	3	1	3	1	10	3	Hi	Non-type	
40	40,5	230,3	0_LoLo	64	M	T4b	N0	1	4	0	3	1	3	1	Lo	Luminal	
41	13,6	18,1	0_LoLo	73	M	T4b	N0	1	4	0	7	1	7	0	Lo	Non-type	
42	45,0	255,8	0_LoLo	82	F	T4	N1	1	4	1	3	1	11	1	Lo	Luminal	
43	28,3	274,3	0_LoLo	74	M	T3a	N0	1	3	1	6	0	36	0	Lo	Luminal	2
44	538,5	211,1	1_3LoHi	70	F	T3b	N2	0	4	0	2	1	2	2	Hi	Non-type	
45	51,4	21,1	0_LoLo	61	F	T3b	N1	1	4	1	4	1	9	1	Lo	Luminal	0
46	537,3	544,1	3_HiHi	67	M	T2	N0	1	2	0	20	0	20	2	Hi	Non-type	
47	57,9	801,2	2_3HiLo	77	M	T2a	N0	0	2	1	2	0	6	1	Lo	Luminal	1
48	223,5	403,3	3_HiHi	84	M	T3a	N0	1	3	0	24	0	24	1	Lo	Non-type	
49	537,0	395,1	3_HiHi	65	M	T2b	N1	0	4	0	24	0	24	2	Hi	Basal	3
50	126,0	415,2	3_HiHi	81	M	T4	N0	1	3	1	6	1	11	3	Hi	Non-type	
51	72,8	184,0	0_LoLo	85	M	T3a	N2	1	4	1	3	1	6	2	Hi	Basal	3
52	128,0	133,3	1_3LoHi	76	M	T3a	N1	0	4	1	6	1	19	1	Lo	Non-type	
53	225,0	294,3	3_HiHi	82	M	T3a	N0	1	3	1	4	0	9	1	Lo	Luminal	
54	28,1	756,3	2_3HiLo	81	F	T3a	N1	0	4	1	5	1	14	1	Lo	Non-type	
55	25,8	110,3	0_LoLo	85	M	T3a	N0	1	3	1	3	1	8	1	Lo	Luminal	
56	82,8	609,1	3_HiHi	65	M	T2b	N0	0	2	0	16	0	16	2	Hi	Luminal	
57	29,2	654,4	2_3HiLo	72	M	T3a	N0	1	3	0	9	0	9	2	Hi	Luminal	
58	51,1	285,5	0_LoLo	47	F	T4	N0	0	3	1	1	1	4	3	Hi	Basal	3
59	182,8	55,3	1_3LoHi	85	M	T4	N1	1	4	0	10	0	10	2	Hi	Basal	1
60	116,1	98,7	1_3LoHi	81	M	T4	N0	1	3	0	9	0	9	1	Lo	Non-type	
61	206,1	1686,7	3_HiHi	77	M	T2a	N0	1	2	0	9	0	9	2	Hi	Luminal	
62	87,5	955,3	3_HiHi	83	M	T2a	N0	1	2	0	7	0	7	1	Lo	Luminal	
63	887,3	1405,5	3_HiHi	87	M	T2b	N0	1	2	0	7	0	7	2	Hi	Non-type	
64	151,1	865,4	3_HiHi	74	M	T3a	N0	0	3	0	5	0	5	3	Hi	Basal	3
65	33,0	243,3	0_LoLo	72	M	T2a	N1	1	4	0	4	0	4	3	Hi	Luminal	1
66	52,1	319,6	2_3HiLo	69	M	T3a	N2	1	4	0	18	1	18	2	Hi	Luminal	2
67	45,7	366,5	2_3HiLo	79	M	T3a	N1	0	4	0	1	1	1	1	Lo	Non-type	

68	27,1	1083,2	2_3HiLo	72	M	T2	N0	1	2	0	8	0	8	0	Lo	Luminal	1
69	9,0	298,1	2_3HiLo	41	F	T4a	N1	0	4	1	27	0	31	3	Hi	Basal	2
70	442,5	1385,0	3_HiHi	85	M	T4a	N0	0	3	0	8	1	8	1	Lo	Basal	3
71	142,4	176,1	1_3LoHi	80	M	T3a	N1	0	4	0	8	1	8	1	Lo	Luminal	0
72	192,7	356,9	3_HiHi	82	F	T3a	N1	0	4	0	4	1	4	0	Lo	Non-type	
73	647,1	106,3	1_3LoHi	54	M	T3a	N0	1	3	1	3	0	24	0	Lo	Luminal	1
74	155,8	1011,9	3_HiHi	66	M	T3a	N2	1	4	1	3	0	17	0	Lo	Luminal	2
75	23,8	174,0	0_LoLo	82	M	T4	N1	1	4	0	9	0	9	1	Lo	Non-type	
76	516,7	195,8	1_3LoHi	79	F	T3a	N0	0	3	1	2	1	3	3	Hi	Basal	3
77	184,1	304,3	3_HiHi	50	M	T4	N0	0	3	1	1	0	9	1	Lo	Luminal	
78	36,2	70,1	0_LoLo	82	F	T3a	N0	1	3	0	1	1	1	2	Hi	Luminal	0
79	9,4	126,9	0_LoLo	79	M	T3a	N0	1	3	0	6	0	6	1	Lo	Luminal	
80	18,7	157,0	0_LoLo	65	M	T4	N2	0	4	0	4	0	4	1	Lo	Luminal	
81	42,5	420,3	2_3HiLo	65	M	T4a	N2	1	4	1	1	1	4	2	Hi	Luminal	1
82	29,1	130,0	0_LoLo	79	M	T3a	N0	0	3	0	4	0	4	1	Lo	Luminal	
83	64,7	77,2	0_LoLo	71	M	T4	N0	1	3	0	3	1	3	0	Lo	Luminal	
84	37,3	837,0	2_3HiLo	75	F	T3a	N2	1	4	1	4	0	4	1	Lo	Luminal	

Supplementary Table S2. Reagents for gene expression assay on UBC lines.

Gene	Cat. no.	Company
**CXCL1**	qHsaCEP0053391	Bio-Rad
**CXCL2**	qHsaCEP0058163	Bio-Rad
**CX3CL1**	qHsaCEP0049180	Bio-Rad
**CXCL8**	qHsaCEP0053894	Bio-Rad
**HPRT1**	4333768T	Applied Biosystems, Thermo Fisher Scientific

Supplementary Table S3: genes defining the PMN and Chemokine signatures.

Neutrophil signature	Chemokine signature	
	Chemokine	Inflammatory functions
ARG1	CCL14	No
CEACAM3	CCL15	No
CEACAM8	CCL16	No
CHI3L1	CCL18	No
CXCR1	CCL19	No
CXCR2	CCL21	No
CYP4F3	CCL23	No
FCGR3B	CCL24	No
FPR2	CCL25	No
HSPA6	CCL27	No
IL1R2	CCL28	No
KCNJ15	CCRL2	No
MME	CXCL12	No
MMP9	CXCL13	No
MMP25	CXCL14	No
OLR1	CXCL17	No
PGLYRP1	CCL1	Yes
S100A8	CCL11	Yes
S100A9	CCL13	Yes
S100P	CCL17	Yes
TNFAIP6	CCL2	Yes
TNFRSF10C	CCL20	Yes
TREM1	CCL22	Yes
VNN3	CCL26	Yes
	CCL3	Yes
	CCL3L1	Yes
	CCL4	Yes
	CCL4L2	Yes
	CCL5	Yes
	CCL7	Yes
	CCL8	Yes
	CKLF	Yes
	CX3CL1	Yes
	CXCL1	Yes
	CXCL10	Yes
	CXCL11	Yes
	CXCL16	Yes
	CXCL2	Yes
	CXCL3	Yes
	CXCL5	Yes
	CXCL6	Yes
	CXCL8	Yes
	CXCL9	Yes
	XCL1	Yes
	XCL2	Yes

Supplementary Table S4. Summary statistics of the CD3 and CD66b as continuous variable, p values by Shapiro-Wilk normality test

	**Min.**	**1st Q**	**Median**	**Mean**	**3rd Q**	**Max.**	**p**
**CD3 area (mm2)**	7.50	73.45	144.26	156.07	233.54	392.99	0.015
**CD3 density (cells/mm2)**	18	204	396	501	646	2020	<0.001
**CD66b area (mm2)**	7.39	72.55	137.37	151.29	222.19	420.72	0.015
**CD66b density (cells/mm2)**	9	32	78	167	183	1049	<0.001
